# *Gynostemma pentaphyllum* polysaccharides ameliorate non-alcoholic steatohepatitis in mice associated with gut microbiota and the TLR2/NLRP3 pathway

**DOI:** 10.3389/fendo.2022.885039

**Published:** 2022-07-22

**Authors:** Si-Ran Yue, Yi-Yun Tan, Lei Zhang, Bao-Jun Zhang, Feng-Yan Jiang, Guang Ji, Bao-Cheng Liu, Rui-Rui Wang

**Affiliations:** ^1^ Shanghai Innovation Center of Traditional Chinese Medicine Health Service, Shanghai University of Traditional Chinese Medicine, Shanghai, China; ^2^ Institute of Digestive Diseases, Longhua Hospital, Shanghai University of Traditional Chinese Medicine, Shanghai, China

**Keywords:** *gynostemma pentaphyllum* polysaccharides, non-alcoholic steatohepatitis, inflammation, gut microbiota, TLR2, NLRP3

## Abstract

Recent studies have revealed the pivotal role of gut microbiota in the progress of liver diseases including non-alcoholic steatohepatitis (NASH). Many natural herbs, such as *Gynostemma pentaphyllum* (GP), have been extensively applied in the prevention of NASH, while the bioactive components and underlying mechanism remain unclear. The aim of this study was to investigate whether the polysaccharides of GP (GPP) have a protective effect on NASH and to explore the potential mechanism underlying these effects. C57BL/6 male mice were fed with a methionine-choline-deficient (MCD) diet for 4 weeks to induce NASH and administered daily oral gavage of sodium carboxymethylcellulose (CMC-Na), low dose of GPP (LGPP), high dose of GPP (HGPP), and polyene phosphatidylcholine capsules (PPC), compared with the methionine-choline-sufficient (MCS) group. Our results showed that the symptoms of hepatic steatosis, hepatocyte ballooning, liver fibrosis, and oxidative stress could be partially recovered through the intervention of GPP with a dose-dependent effect. Furthermore, gut microbiome sequencing revealed that HGPP altered the composition of gut microbiota, mainly characterized by the enrichment of genera including *Akkermansia*, *Lactobacillus*, and *A2*. Moreover, hepatic transcriptome analysis indicated that the anti-inflammatory effect of HGPP might be associated with toll-like receptor (TLR) and nod-like receptor (NLR) signaling pathways. HGPP could inhibit the expression of TLR2 and downregulate the expression of the NLRP3 inflammasome, as well as the pro-inflammatory cytokine tumor necrosis factor (TNF)-α and interleukin (IL)-1β. In summary, GPP could ameliorate NASH possibly mediated *via* the modulation of gut microbiota and the TLR2/NLRP3 signaling pathway, indicating that GPP could be tested as a prebiotic agent in the prevention of NASH.

## Introduction

Non-alcoholic steatohepatitis (NASH), the aggressive form of non-alcoholic fatty liver disease (NAFLD), is characterized by liver steatosis, inflammation, and hepatocyte damage with different degrees of fibrosis (1–3). The global prevalence of NAFLD is approximately 25%, and the pooled overall NASH prevalence estimate among NAFLD patients is 59.10% (4). Emerging evidence suggests that the trigger of inflammation plays a key role in the progression from isolated steatosis state to NASH (2) and caused an increase in the risks of liver cirrhosis and hepatocellular carcinoma (HCC) (5, 6). However, limited medications are available by far to meet the increasing disease burden of NASH, and first-line therapy still mainly relies on lifestyle interventions including diet, exercise, and weight loss (7). Therefore, developing new therapeutic agents for preventing and treating NASH will be of high significance.

Recently, dysbiosis of the gut microbiota has been proposed to be causally and mechanistically associated with many liver diseases, including NASH (8). Studies in germ-free mice demonstrated that the gut microbiota is a primary causative agent of liver steatosis and NAFLD (9, 10). The gut microbiota and its metabolites influence the liver through the gut–liver axis, which plays an important role in the pathogenesis of hepatic lipid metabolism and inflammation (11). An essential component of the gut–liver axis may be associated with the role of the gut microbiota in the sphingolipid synthesis pathway in hepatic sinusoidal endothelial cells (12). Dysfunction of the intestinal epithelial barrier can markedly contribute to the increased translocation of gut microbial products and aggravate hepatic inflammation in NASH (13). Moreover, the serum concentration of endotoxin lipopolysaccharide (LPS), a component of gram-negative bacteria, was found to be increased in NASH (14). The intestine-derived bacterial product peptidoglycan (PGN) and LPS could enter the portal vein *via* the impaired gut barrier, thereby inducing an inflammatory response in the liver mediated by TLRs (15–17). For instance, TLR2, the recognizer of PGN, is required in the activation of the nod-like receptor protein 3 (NLRP3) inflammasome and the secretion of interleukin (IL)-1 which results in progression to NASH (18). Therefore, it is necessary to understand the contribution of the gut microbiota in the pathogenesis and improvement in NASH.

Many herbal remedies used in traditional Chinese medicine (TCM) exert hepatoprotective, anti-inflammatory, and anti-fibrotic effects in the intervention of NASH (19–21). *Gynostemma pentaphyllum* (GP), named “Jiao-Gu-Lan” in Chinese, is an edible and medicinal herbaceous climbing plant from the family Cucurbitaceae and has long been used as anti-inflammation TCM in China (22). The active component of GP has been mainly considered as saponins, while recently the purified or crude polysaccharides were also reported as the active ingredients of GP (23). The polysaccharides are enzymatically indigestible in the animal upper gastrointestinal tract until they reach the colon and are digested by the symbiotic bacteria; therefore, increasing attention has been paid to the effects of polysaccharides associated with the gut microflora (24, 25). Previous studies have indicated that several TCM polysaccharides from *Eucommia ulmoides* (26) and *Hirsutella sinensis* (27) could play an anti-inflammatory role by enhancing the growth of specific gut bacteria and the production of microbiota-derived metabolites (28–30). However, the effect of GP polysaccharides (GPP) in the treatment of NASH and the possible molecular mechanisms still need to be explored.

In the current study, the anti-NASH effects of GPP were evaluated in the murine MCD model of NASH. Gut microbiome and hepatic transcriptomics analyses were applied to reveal the underlying mechanism. It has been found that a high dose of GPP (HGPP) exerted prevention effect on MCD-induced C57BL/6 mice through the modulation of the gut microbiota and TLR2/NLRP3 signaling pathway.

## Materials and methods

### GPP preparation

GP was extracted three times with 80°C boiled water for 2 h each time. The concentrated filtrate was added into the AB-8 macroporous resins and eluted with water. GPP was obtained by lyophilizing for 48 h with a freeze dryer.

### Determination of molecular weight and monosaccharide composition

The molecular weight of GPP fractions was determined by high-performance gel permeation chromatography (HPGPC) equipped with a Shimadzu HPLC system (Shimadzu, Kyoto, Japan) fitted with one BRT105-104-102 (8 × 300 mm) (BoRui Saccharide, Yangzhou, China) and a RID-10A detector (Shimadzu, Kyoto, Japan). Briefly, 5 mg/ml of GPP sample was injected and analyzed on the HPGPC system at a flow rate of 0.6 ml/min with 0.05 M NACl at 40°C and monitored with the differential refractive index (dRI). The sample was filtered using a 0.22-μm filter before injection.

The monosaccharide composition was analyzed by ion-exchange chromatography (IC) using a Dionex™ Carbopac™ PA20 column (ICS5000, Thermo Fisher, Waltham, USA). Briefly, 10 mg of GPP sample was dissolved in 10 ml of 3 M trifluoroacetic acid (TFA) at 120°C for 3 h. After evaporation, the solution was dissolved in 10 ml of H_2_O, 100 μl was taken, and 900 μl of ddH_2_O was added. After 12,000 rpm centrifugation for 5 min, the supernatant was applied to HPIC using H_2_O, 15 mM NaOH, and 100 mM NaOAc as the mobile phase with a flow rate of 0.3 ml/min.

### Mouse model

Male C57BL/6 9-week-old mice (n = 35) were purchased from the Shanghai Model Organisms Center (Shanghai, China). The mice were maintained in a specific pathogen-free (SPF) grade and under standard conditions of illumination (12-h light/dark cycle). After 1 week of adaptive feeding, the mice were randomly divided into five groups. The control group was fed with a methionine and choline-sufficient (MCS) diet (#519581, Dyets) and received intragastric administration of 0.5% sodium carboxymethylcellulose (CMC-Na) at the same time for 4 weeks, and the model group was fed with an MCD diet (#519580, Dyets) and received intragastric administration of 0.5% CMC-Na at the same time for 4 weeks. In the GPP treatment groups, the mice were fed with an MCD diet and received intragastric administration of either at 150 mg/kg of low dose (LGPP) or 300 mg/kg of high dose (HGPP). In the positive treatment group, the mice were fed with an MCD diet and received intragastric administration of 120 mg/kg of polyene phosphatidylcholine capsules (PPCs). The GPP and PPC powders were dissolved in 0.5% CMC-Na for intragastric administration, and an equal volume of 0.5% CMC-Na vehicle was given to the MCS and MCD mice. GPP (purity: 98.93%) was purchased from Shanghai Winherb Medical Technology (#200607, Shanghai, China). PPCs were purchased from Sanofi (#H20059010, Beijing, China).

After 4 weeks, all mice were sacrificed by cranio-cervical dislocation. The blood sample was collected by extracting the eyeball, and serum was separated after centrifugation (2,500 rpm, 15 min) of the blood for biochemical analyses. The livers were immediately removed, weighed, and washed with PBS. Following evisceration, the liver samples were dissected from the right lobe of the liver. All samples were collected and stored at -80°C for long-term storage.

All procedures performed in the study involving animals were in accordance with the ethical standards of and protocol approved by the Shanghai Model Organisms Center (Shanghai, China).

### Hepatic and serum biochemical analyses

Hepatic total cholesterol (TC, #A111-1-1), triglycerides (TG, #A110-1-1), superoxide dismutase (SOD, #A001-3-2), malondialdehyde (MDA, #A003-1-2), and hydroxyproline (HYP, #A030-2-1) were measured according to the instruction, and all kits were purchased from Nanjing Jiancheng Bioengineering Institute (Nanjing, China). Serum TC, TG, low-density lipoprotein cholesterol (LDL-C), high-density lipoprotein cholesterol (HDL-C), alanine transaminase (ALT), and aspartate aminotransferase (AST) were measured using automated clinical chemistry analyzer (7020, Hitachi, Japan) in the Center for Drug Safety Evaluation and Research, Shanghai University of Traditional Chinese Medicine (Shanghai, China).

### Histological analysis

Liver tissues from the mice were fixed in 4% polyformaldehyde and embedded in paraffin wax. Sections were cut and stained with hematoxylin and eosin (H&E) and Sirius Red. Oil Red O staining was performed on frozen liver specimens. For the histological diagnosis of NASH, the NAFLD Activity Score (NAS) was evaluated by a pathologist using standards as previously described and NAS ≥5 served as a substitute indicator for the histological diagnosis of NASH (31). Images were quantified using Image-Pro Plus software 6.0 (Rockville, USA), three randomly chosen fields per sample. The percentages of Oil Red O staining and Sirius Red staining were calculated as the average e area of Oil Red O staining and Sirius Red staining relative to the total analyzed area.

### Gut microbiome sequencing

To determine the structure and function profile of the gut microbial community, high-throughput sequencing was used to sequence the 16S ribosomal RNA gene v3–v4 regions of the gut microbiota in mouse colon contents. The genomic DNA of the gut microbiota was extracted using the E.Z.N.A.^®^ Stool DNA Kit (#D4015-04, Omega Bio-tek, Norcross, USA) from colon contents according to the manufacturer’s instructions. The concentration and purity of extracted DNA were determined with NanoDrop 2000 (Thermo Scientific, Waltham, USA). The DNA extract quality was checked on 1% agarose gel. The v3–v4 regions of the 16S rRNA gene were amplified using 338F (5′ ACTCCTACGGGAGGCAGCAG 3′) and 806R (5′ GGACTACHVGGGTWTCTAAT 3′) primers. The multiplexed amplicons were purified using the AxyPrep DNA Gel Extraction Kit (#AP-GX-250G, Axygen Biosciences, Union City, USA). Amplicon sequencing was performed with Illumina MiSeq PE3000 (Illumina, San Diego, USA) at HonsunBio Biotechnology Co., Ltd. (Shanghai, China). The QIIME platform provided a 16S rRNA gene−amplicon−based analysis (https://www.qiime.org). The sequences allowed sorting of the reads into operational taxonomic units (OTUs) by clustering 97% sequence similarity and aligned using the SILVA database. Taxonomic classification was conducted according to various taxonomic ranks (phylum, order, class, family, genus, and species). The percentage of each bacterial species was analyzed and plotted by the R programming language. Sequence data associated with this project have been submitted to NCBI Sequence Read Archive (Accession Number: SRP361247).

### Transcriptomics

Total RNA was extracted from livers of MCS, MCD, and HGPP groups (n = 3). Liver samples were lysed using TRIzol reagent (#15596018, Ambion, Austin, USA), and RNA was isolated with chloroform, followed by isopropanol precipitation and 75% ethanol washing. The RNA was redissolved in RNase-free water, and integrity and purity were assessed using a NanoDrop spectrophotometer (Thermo Scientific, Waltham, USA). RNA-sequencing library preparation was generated using 3 μg of RNA as input material. A sequencing library was generated on the NovaSeq 6000 platform (Illumina, San Diego, USA) by Personalbio Technology (Shanghai, China). After sequencing, the reads were preprocessed with Cutadapt and removed with an alignment quality (<Q20). Gene read counts were estimated with HTSeq (0.9.1) statistics, and then differential expression analysis was performed using DESeq (1.30.0) with a threshold of |log2FoldChange| >1 and *P* value < 0.05. Heatmaps of gene expression were generated using the R language pheatmap (1.0.8) software package and were generated based on Fragments per Kilobase of exon model per Million mapped fragments (FPKM). Gene Ontology (GO) enrichment was performed using topGO based on the hypergeometric distribution method with *P* value < 0.05. GO biological processes (BPs) were chosen for displaying the clustering results. Kyoto Encyclopedia of Genes and Genomes (KEGG) enrichment was carried out by clusterProfiler (3.4.4) software, focusing on the significant enrichment pathway with *P* value < 0.05. Gene Set Enrichment Analysis (GSEA) for KEGG pathways was conducted using the GSEA website, and the gene sets were obtained from the MSigDB database (http://software.broadinstitute.org/gsea/msigdb). The GSEA enrichment plotted by R language gseaplot2 (1.2.0) offered the visualization by results with |NES|>1 and *P* adjust<0.05. Data were uploaded to the NCBI GEO database (GSE205974) and analyzed on the online platform Personalbio GenesCloud (https://www.genescloud.cn).

### Quantitative real-time polymerase chain reaction

Total RNA was extracted from homogenizing liver tissue, and cDNA was synthesized using HiScript II Q RT SuperMix for qPCR Kit (Vazyme, Nanjing, China). qPCR was performed by using a Luna Universal qPCR Master Mix (#M3003L, New England Biolabs, Ipswich, USA), and all the primers are presented in **Table S1**. GAPDH was used as the endogenous reference control. All primers were purchased from Sangon Biotech (Shanghai, China). The data were analyzed by 2^-ΔΔCt^.

### Western blotting

This section was performed in accordance with the former studies. Briefly, proteins from liver tissues were extracted by sonication in RIPA lysis buffer with phenylmethanesulfonylfluoride. After centrifugation at 12,000 rpm for 10 min at 4°C, the supernatant of the lysate was collected and the protein concentration was detected by the BCA method. The proteins of samples were fractionated through 8% SDS-PAGE and transferred onto NC membranes. After being blocked with 5% non-fat milk for 1 h at room temperature, the membranes were incubated with different primary antibodies overnight at 4°C. After washing with TBST three times (10 min each), the blots were incubated with secondary antibodies for 1 h. Finally, the signal was visualized using ECL reagent. Pro-Caspase-1 (#YT0652, 1:1,000), Caspase-1 (#YC0002, 1:1,000), β-actin (#YT0099, 1:5,000), and secondary antibody HRP* Goat Anti Rabbit IgG(H+L) (#RS0002, 1:10,000) antibody were purchased from ImmunoWay Biotechnology (Plano, USA). Western blot quantification was analyzed densitometrically using ImageJ software and normalized to β-actin.

### Immunohistochemistry

Liver tissues were embedded in paraffin, and slides were cut from the paraffin tissue. Slides were heated with sodium citrate for antigen retrieval and then incubated with primary antibodies including TLR2 (#GB11554, 1:300, Servicebio, Wuhan, China), NLRP3 (#YT5382, 1:100, ImmunoWay Biotechnology), ASC (#YT0365, 1:200, ImmunoWay Biotechnology), Caspase-1 (#GB11383, 1:500, Servicebio), and IL-1β (#GB11113, 1:250, Servicebio) overnight at 4°C. Secondary antibody HRP-conjugated Goat Anti-Rabbit IgG(H+L) (#GB23303, 1:200, Servicebio) was incubated for 50 min at room temperature. After PBS washing, peroxidase substrate DAB (#K5007, DaKo, Copenhagen, Denmark) was used as chromogen. The slices were stained, differentiated, and sealed before visualizing and taking photos under the microscope. Images of IHC were quantified using Image-Pro Plus 6.0 and assessed based on the sum value of integrated optical density (IOD, Area × Intensity) (32, 33). The average numbers of IOD were counted from three randomly chosen fields.

### Enzyme-linked immunosorbent assay

Mouse lipopolysaccharide binding protein (LBP) ELISA kit (#PCDBN0177) and mouse IL-1β ELISA kit (#PCDBM0158) were provided by PC Biotech (Shanghai, China). The serum levels of IL-1β and LBP were determined using commercial kits according to the instruction manual based on sandwich ELISA.

### Statistical analysis

Data are presented as median and interquartile range (IQR) using violin plots. Comparisons among multiple groups were performed using one-way analysis of variance (ANOVA) for normal distribution and Kruskal–Wallis test for non-normal distribution. One-way ANOVA was followed by a least significant difference (LSD) test. Comparisons between two groups were performed using unpaired t-test for normal distribution and Mann–Whitney test for non-normal distribution. *P* < 0.05 was considered statistically significant. Statistical tests used to compare conditions are indicated in figure legends. The violin plots indicated lower quartiles, median, and higher quartiles, from bottom to top. All statistical analyses were performed using GraphPad Prism 8.3.0 software (San Diego, USA) or SPSS 25.0 software (Chicago, USA).

## Results

### The analysis of GPP compositions

**Figure 1:**
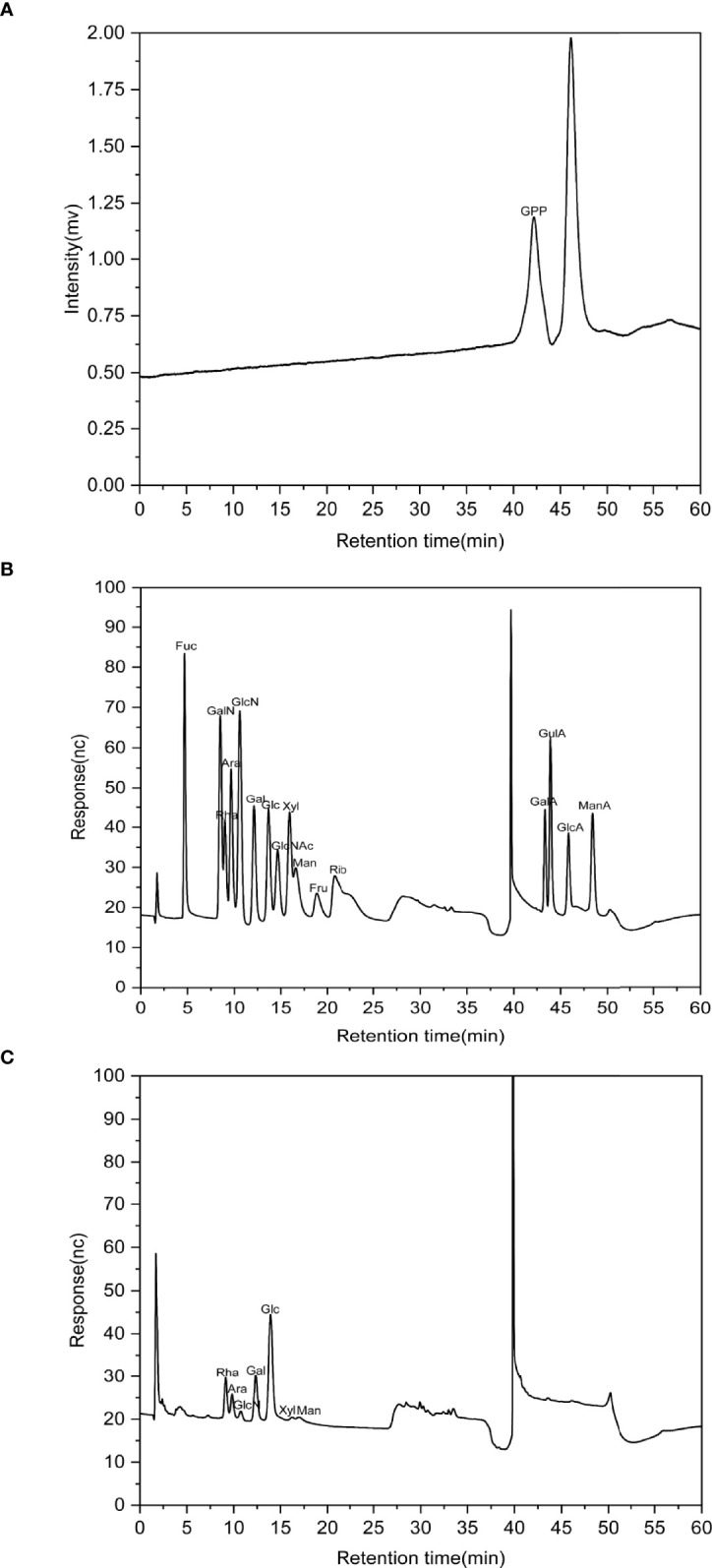
The characterization of *Gynostemma pentaphyllum* polysaccharides (GPP). **(A)** High-performance gel permeation chromatography (HPGPC) chromatogram of GPP. **(B)** Ion-exchange (IC) chromatograms of standard monosaccharides and **(C)** component monosaccharides of GPP.

The HPGPC profile showed that the molecular weight of GPP was 1.08 kDa (**Figure 1A**). IC analysis showed the presence of rhamnose (Rha), arabinose (Ara), glucosamine hydrochloride (GlcN), galactose (Gal), glucose (Glc), xylose (Xyl), and mannose (Man) in the ratio of 0.208:0.069:0.015:0.186:0.467:0.018:0.037 for GPP (**Figures 1B, C**).

### Effects of GPP on MCD-induced NASH mice

**Figure 2:**
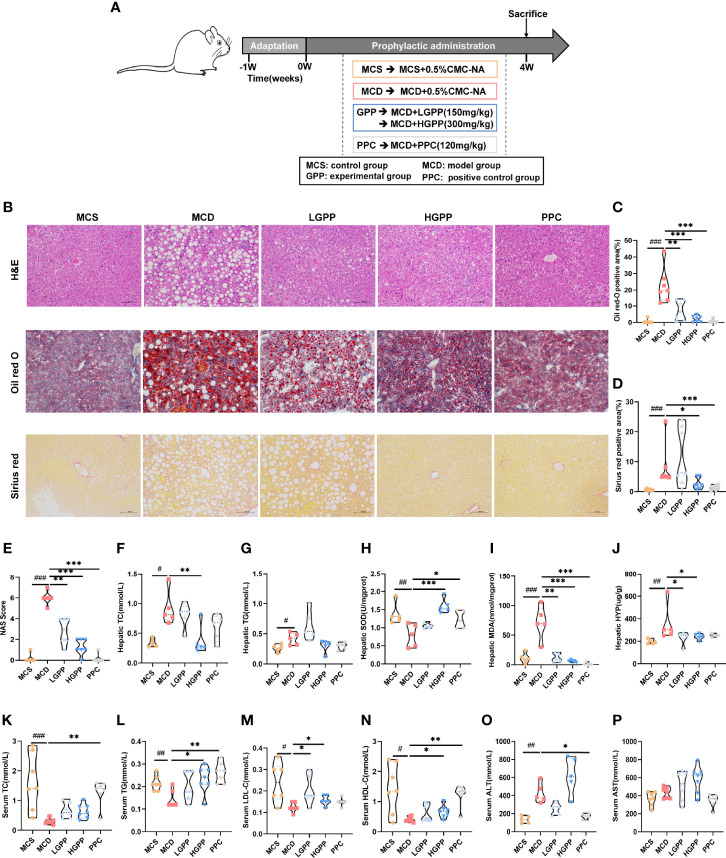
GPP alleviated methionine-choline-deficient (MCD)-induced non-alcoholic steatohepatitis (NASH) (n = 5–7). **(A)** Study design. **(B)** Representative photomicrographs of liver tissue with H&E (magnification, ×200, 100 μm) and Oil Red O staining (magnification, ×200, 100 μm) and Sirius Red staining for liver sections (magnification, ×200, 100 μm). **(C)** Histomorphometric analysis of the positive area with Oil Red O staining and **(D)** Sirius Red staining. **(E)** Non-alcoholic fatty liver disease (NAFLD) Activity Score (NAS). **(F–J)** Hepatic total cholesterol, triglycerides, superoxide dismutase, malondialdehyde, and hydroxyproline (TC, TG, SOD, MDA, and HYP, respectively). **(K–P)** Serum TC, TG, low- and high-density-lipoprotein cholesterol, alanine transaminase, and aspartate aminotransferase (LDL-C, HDL-C, ALT, and AST, respectively). Data are expressed as median and interquartile range (IQR). *P < 0.05, **P < 0.01 and ***P < 0.001 vs. the MCD group; ^#^ *P* < 0.05, ^##^ *P* < 0.01, and ^###^ *P* < 0.001 vs. MCS group. One-way ANOVA test followed by a least significant difference (LSD) **(H, P)**, Kruskal–Wallis test **(C–G, I–O)**. MCS, methionine-choline-sufficient; MCD, methionine-choline-deficient; LGPP, low dose of GPP; HGPP, high dose of GPP; PPC, polyene phosphatidylcholine capsules.

To explore the potential effects of GPP on NASH, an animal experiment was conducted as shown in **Figure 2A**. Pathological results showed the phenotype of NASH including ballooning degeneration, steatosis, lobular inflammation, and fibrosis induced by MCD diet. GPP and PPC improved the hepatic steatosis, while HGPP showed a similar efficacy as the PPC group based on the measurement of positive area staining and NAS score (**Figures 2B–E**). Regarding hepatic lipid level, the MCD group showed significantly increased levels of hepatic TC and TG, while the HGPP group showed a significant decreased level of hepatic TC (**Figures 2F, G**). MCD also induced oxidative damage in the liver as reflected by the SOD and MDA indexes. GPP and PPC had a significant ameliorative effect on the hepatic MDA, while HGPP and PPC presented a better effect on the hepatic SOD (**Figures 2H, I**). Meanwhile, HYP assay revealed that both LGPP and HGPP could significantly decrease the liver collagen content, while PPC showed no statistical difference (**Figure 2J**). Furthermore, MCD diet significantly decreased the levels of serum TC, TG, LDL-C and HDL-C, which could be partially recovered by different doses of GPP and PPC, respectively (**Figures 2K–N**). In contrast to the significant downregulation in the PPC group, the level of ALT showed a downward tendency in the LGPP group (*P* = 0.13) but an upward tendency in the HGPP group (*P* = 0.07). Moreover, the differences were not statistically significant in the level of AST among five groups (**Figures 2O, P**). These results indicated that GPP could ameliorate the MCD-induced NASH phenotype including hepatic steatosis, oxidative stress, and fibrosis in part. In addition, HGPP showed superior efficacy when compared with LGPP, so the further mechanistical exploration was mainly focused on the HGPP group.

### Composition of the intestinal flora modified by HGPP treatment

**Figure 3:**
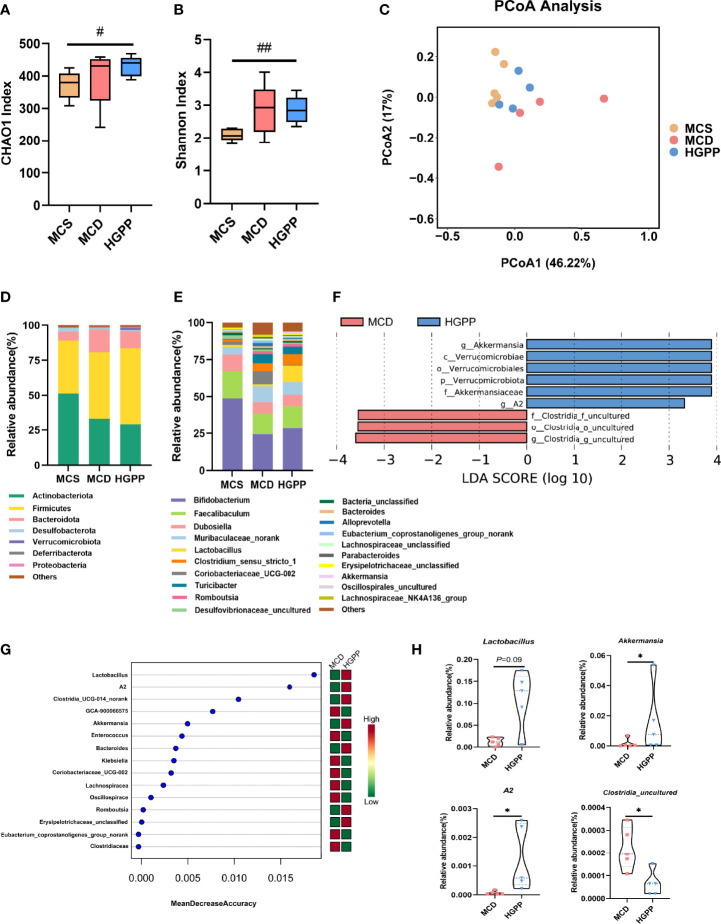
HGPP changed the composition of the gut microbiota. **(A)** The alpha diversity analysis, including CHAO1 diversity and **(B)** Shannon diversity (n = 4–5). **(C)** Principal coordinate analysis (PCoA) based on the Bray–Curtis of mouse colon content microbiota in MCS, MCD, and HGPP groups (n = 4–5). **(D)** Changes in colonic bacterial composition (relative abundance) at the phylum level (n = 5) and **(E)** genus level (n = 5). **(F)** Linear discriminant analysis (LDA) effect size (LEfSe) analysis for differential abundant taxa detected between MCD and HGPP groups based on the abundance profiles. Threshold parameters were set as *P* = 0.05 for the Mann–Whitney U test. LDA score > 2.0 (n = 5). **(G)** Top 15 bacterial genera influencing mean decreased accuracy of random forest classification between MCD and HGPP colonic microbiota (n = 5). **(H)** The relative abundance of *Lactobacillus*, *Akkermansia*, *A2*, and *Clostridia_uncultured* between MCD and HGPP groups (n = 5). Data are expressed as median and interquartile range (IQR)Please revise: *P <0.05, vs. the MCD group; ^#^ *P* <0.05 and ^##^ *P* < 0.01 vs. the MCS group. Kruskal–Wallis test **(A, B),** Mann–Whitney U test **(H)**.

The gut microbiota composition was profiled by sequencing the v3–v4 areas of the 16S rRNA gene. The alpha-diversity reflected by Chao1 and Shannon indexes showed no statistical difference between MCD and HGPP groups (**Figures 3A, B**). Principal coordinate analysis (PCoA) based on Bray–Curtis distance showed a clear separation between three groups, and the HGPP group clustered closer to the MCS group. Differences in analysis of similarity (ANOSIM) were significant for the three groups (*P* = 0.002, ANOSIM *R* = 0.5341) (**Figure 3C**). In addition, we observed significant changes in the relative abundance at the phylum and top 20 genus levels (**Figures 3D, E**). LEfSe analysis between MCD and HGPP groups showed that *Akkermansia* and *A2* were enriched in the HGPP group, while *Clostridia_uncultured* was enriched in the MCD group at the genus level (**Figure 3F**). Random forest ranked the top 15 genera which contribute to the discrimination of MCD and HGPP groups, the top 5 including *Lactobacillus*, *A2*, *Clostridia_UCG-014_norank*, *GCA-900066575*, and *Akkermansia*. (**Figure 3G**). The relative abundances of *Lactobacillus*, *Akkermansia*, *A2*, and *Clostridia_uncultured* were further compared between the MCD and HGPP groups. There was a trend (*P* = 0.09) for higher relative abundance of *Lactobacillus* in the HGPP group. Moreover, HGPP was found to have a significantly increased relative abundance of *Akkermansia* and *A2* and a significantly decreased relative abundance of *Clostridia_uncultured* compared to MCD (**Figure 3H**).

### HGPP regulated hepatic gene expression profiles in MCD-fed mice

**Figure 4:**
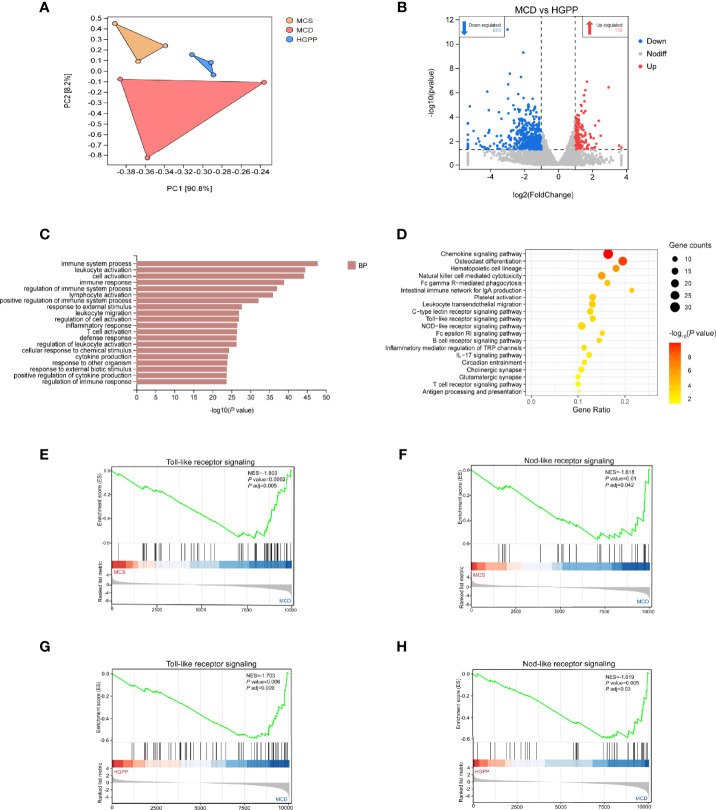
HGPP caused a shift in gene expression in mice liver (n = 3). **(A)** Principal component analysis (PCA) of transcriptional profiling of the mouse liver tissues among three groups. **(B)** Screening different genes by volcano plot (|log_2_Foldchange|>1, *P* value <0.05) between MCD vs. HGPP group. **(C)** Gene Ontology (GO) pathway enrichment analysis of top 20 pathways between the MCD and HGPP groups. **(D)** Kyoto Encyclopedia of Genes and Genomes (KEGG) pathway enrichment analysis of top 20 pathways between the MCD and HGPP groups. **(E)** Gene Set Enrichment Analysis (GSEA) of toll-like receptor (TLR) signaling between the MCS and MCD groups. **(F)** GSEA of nod-like receptor (NLR) signaling between the MCS and MCD groups. **(G)** GSEA of TLR signaling between the MCD and HGPP groups. **(H)** GSEA of NLR signaling between the MCD and HGPP groups.

To further understand the potential mechanism underlying the anti-NASH effect of HGPP, the hepatic transcriptional profiles were characterized among different groups of mice. First of all, principal component analysis (PCA) showed a distinct separation among MCS, MCD, and HGPP groups. HGPP treatment resulted in a clear separation in gene expression in the PC2 context of MCD (**Figure 4A**). Volcano plots showed 2,282 genes between the MCS and MCD groups (1,622 upregulated genes, 660 downregulated genes), 1,232 genes between the MCS and HGPP groups (805 upregulated genes, 427 downregulated genes), and 755 genes between the MCD and HGPP groups (132 upregulated genes, 623 downregulated genes) (**Figures S3A, B**, **Figure 4B**). Venn diagrams depicted the number of overlapping and different DEGs for three group comparisons (**Figures S3C–E**). GO enrichment analysis revealed the top 20 significantly enriched GO terms in the biological process, such as regulation of immune system, leukocyte activation, cell activation, inflammatory response, T cell activation, and cytokine production, among others, between the MCD and HGPP groups (**Figure 4C**). Several KEGG signaling pathways, including chemokine signaling pathway, osteoclast differentiation, platelet activation, leukocyte transendothelial migration, toll-like receptor (TLR) signaling pathway, and nod-like receptor (NLR) signaling pathway, were widely enriched between the MCD and HGPP groups (**Figure 4D**). To be able to systematize the gene expression results, we performed GSEA based on all annotated genes to determine the important signaling pathways. In total, there were 40 pathways with |NES|>1 and *P* adjust < 0.05 between the MCS and MCD groups, and there were 42 pathways with |NES|>1 and *P* adjust < 0.05 between the MCD and HGPP groups (**Tables S9, 10**). The overlapping KEGG pathways between MCS vs. MCD and MCD vs. HGPP were screened, including natural killer cell-mediated cytotoxicity, hematopoietic cell lineage, TLR signaling pathway, NLR signaling pathway, B-cell receptor signaling pathway, Fc epsilon RI signaling pathway, and leukocyte transendothelial migration. A previous study has shown that transcriptional upregulation of the inflammatory pathway is typically initiated by pattern recognition receptors (PPRs) such as TLRs and NLRs (34). MCD consistently led to the activation of the TLR and NLR signaling pathways, showing the importance of these two pathways in the development of a murine MCD model of NASH (**Figures 4E, F**). This result was also found between the MCD and HGPP groups (**Figures 4G, H**). As in the analysis above, the liver gene expression profiles reflected altered gene expressions and signaling pathways when HGPP compared to MCD. The possible mechanism was associated with regulating TLR and NLR signaling pathways.

### HGPP modulated the TLR2/NLRP3 signaling pathway in the liver of MCD mice

**Figure 5:**
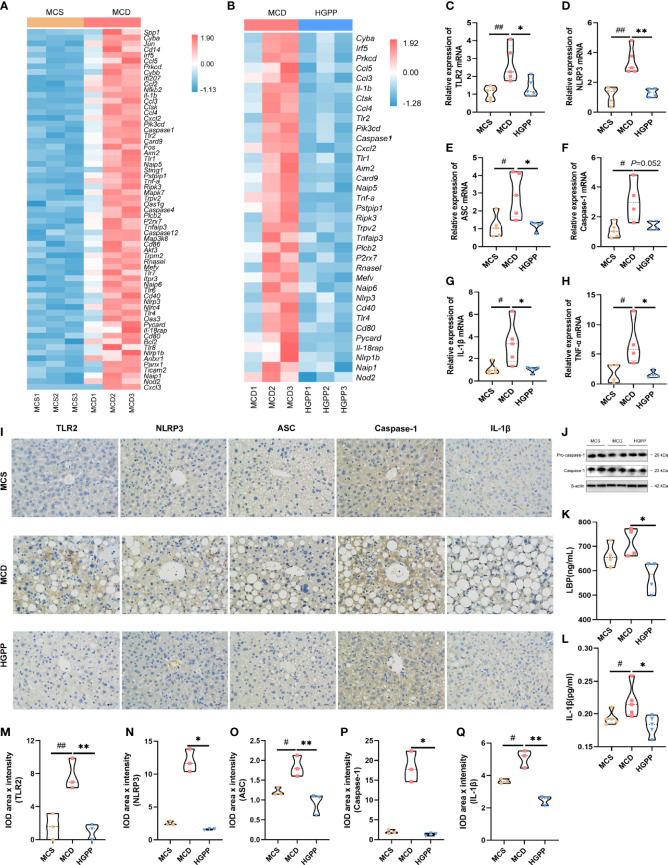
The potential mechanism of the HGPP at the molecular level. **(A)** Heatmap of differential gene expression in TLR and NLR signaling pathways between the MCD and MCS groups. **(B)** Heatmap of differential gene expression in TLR and NLR signaling pathways between the MCD and HGPP groups. **(C–H)** The relative TLR2, NLRP3, ASC, Caspase-1, IL-1b, and TNF-α mRNA expression levels (n = 4-5). **(I)** Immunohistochemical assay of TLR2, NLRP3, ASC, Caspase-1, and IL-1β in hepatic tissue (magnification, ×400, 100 μm) (n = 3). **(J)** Pro-caspase-1 and Caspase-1 expression was evaluated by Western blotting in liver samples. **(K)** The level of LBP in blood serum was tested by enzyme-linked immunosorbent assay (ELISA) (n = 4). **(L)** The level of IL-1β in blood serum was tested by ELISA (n = 5). **(M–Q)** Immunohistochemical images were quantified for integrated optical density (IOD, Area × Intensity) (n = 3). The 2^−△△Ct^ method was used to analyze relative gene expression levels. Data are expressed as median and interquartile range (IQR). *P <0.05 and **P <0.01 vs. the MCD group; ^#^ *P* <0.05 and ^##^ *P* < 0.01 vs. the MCS group. One-way ANOVA test followed by a least significant difference (LSD) **(C, K–M, O, Q)**; Kruskal–Wallis test **(D–H, N, P)**.

To investigate whether the anti-inflammation effect of HGPP treatment impacts TLRs and NLRs on NASH, we examined the potential molecular mechanism. Heatmaps of gene expression (FPKM) showed that compared with that in the MCS group, the expressions of TLR genes (Tlr1, Tlr2, Tlr4, Tlr6, Tlr7, Tlr8) (35), inflammasome activation genes (Nlrp1b, Nlrp3, Nlrc4, Naip1, Naip5, Naip6, Nod2, Card9, Aim2, Caspase1, Caspase4, Caspase12, etc.) (36–39), pro-inflammatory genes (Il-1β, Il-18rap, Tnf-α, etc.) (35), and immune-related genes (Cd14, Cd40, Cd80, Cd86, etc.) (40, 41) were upregulated in the MCD group. Conversely, the expressions of genes including Tlr1, Tlr2, Tlr4, Nlrp1b, Nlrp3, Naip1, Naip5, Naip6, Aim2, Caspase1, Nod2, Card9, Il-1β, Il-18rap, Tnf-α, Cd40, and Cd80 were downregulated from mice treated with HGPP (**Figures 5A, B**). Further qPCR revealed that the expressions of TLR2, NLRP3, ASC, IL-1β, and TNF-α genes enriched in TLR and NLR signaling pathways were significantly downregulated in HGPP compared to the MCD group (**Figures 5C–H**). Immunohistochemical staining and quantification results for TLR2, NLRP3, ASC, Caspase-1, and IL-1β showed that the number of IOD was decreased in HGPP-treated mice (**Figures 5I, M–Q**). The expressions of Pro-caspase-1 and Caspase-1 genes in the livers of the HGPP group were verified by Western blotting (**Figure 5J**, **Figure S4**). We further investigated the serum level of LBP, and HGPP significantly reduced the LBP compared to the MCD group (**Figure 5K**). The serum level of IL-1β was significantly decreased in HGPP treatment (**Figure 5L**). The IHC quantification revealed the decreased IOD of TLR2, NLRP2, ASC, Caspase-1, and IL-1b in HGPP when compared with MCD (**Figures 5M–Q**). These results suggested that HGPP treatment exerts an anti-inflammation effect involved in the TLR2/NLRP3 signaling pathway.

## Discussion

NASH is one of the most common serious liver diseases, and its comorbidities are associated with the leading cause of death, while there is no proven medical treatment for NASH (42). Herbal medicines with pleiotropic actions may offer the possibility of improving NASH effectively (43). In our study, we found that GPP could ameliorate the MCD-induced NASH in a dose–effect manner. It is also found that GPP could improve the gut dysbiosis and inflammation response, which may suppress the activation of the TLR2/NLRP3 signaling pathway.

Our results showed that different doses of GPP can alleviate the degree of steatosis, hepatocyte ballooning, and liver fibrosis pathologically in MCD-fed mice. Previous studies have found that Fuzhenghuayu decoction and Jiangzhi granule, with GP as their main ingredient, could ameliorate hepatic fibrosis and steatohepatitis *in vitro* and *in vivo* (44, 45). Moreover, the compound of Gypenosides LXXV could alleviate NASH by downregulating the inflammation and hepatic fibrosis markers such as TNF-α, IL-1β, and collagen 1 (46). In our study, we showed that GPP is effective in the prevention of steatohepatitis in a murine MCD model of NASH with dose–effect. Furthermore, the HGPP is comparable to PPC on some indexes such as hepatic steatosis, oxidative stress, and fibrosis. Both LGPP and HGPP showed to be more effective on liver fibrosis measured by hepatic HYP than PPC. We found that PPC exerted better amelioration in the level of serum TC and ALT when compared with LGPP and HGPP. Notably, the change trend of ALT differed between the LGPP and HGPP groups, indicating that the hepatotoxicity might be caused by GPP in a murine model of NASH. Therefore, more preclinical and experimental studies are needed to confirm the long-term safety and efficacy outcomes of GPP. Our results proposed that GPP may act as a potential agent in the precaution of NASH.

The effect of polysaccharides from different kinds of plants are highly associated with gut microbiota (47–50). In the colon, fermented polysaccharides are degraded by gut microbes to produce natural bioactive products; meanwhile, the bacteria which can utilize polysaccharides will prosper in return (51). In our study, we found that *Akkermansia*, *Lactobacillus*, and *A2* are the best responders to GPP. *Akkermansia* is a potential probiotics which could lead to improvements in intestinal barrier dysfunction, inflammatory response, and hepatic injury (52–54). As the most commonly used probiotics, the members of *Lactobacillus* have been shown to be anti-inflammatory and protective of intestinal barrier function (55). Furthermore, *Akkermansia* can form an alliance with *Lactobacillus* before the colonization, which increased the abundance of *Akkermansia*, and this ally shows the protective effects and the modulated composition of the gut microbiota (56). Another enriched genus in the HGPP group, *A2*, belongs to the *Lachnospiraceae* family which contains many butyrate producers (57). Meanwhile, serum LPS-binding protein (LBP), which reflects the endotoxin load (58), was also decreased in the HGPP group. Based on the above results, we speculated that HGPP might benefit NASH by altering the composition of gut microbiota.

In addition, transcriptome analysis revealed that the TLR and NLR signaling pathways are candidate pathways in the improvement of NASH by HGPP. TLR2, which plays an important role in the formation the NLRP3/ASC/pro-caspase-1 complex (18, 59), is indicated as a potential key mediator between gut bacteria and inflammation (60). It was found that the oral administration of *Lactobacillus plantarum* can lead to the inhibition of TLR2 in a murine high-fat-diet (HFD) model of obesity (61). Besides, studies performed on TLR2-deficient mice demonstrated that TLR2 could sense gut microbial ligands that can induce remote signaling in the liver endothelium (62). PGN, the bacterial pattern molecule, could signal *via* TLR2 (63) and possess diverse antigenic activities (64). Then, pro*-*caspase*-*1 activates caspase-1 and further orchestrates IL-1β secretion (65). Meanwhile, upregulation of TLR2 signaling could activate the NLRP3 inflammasome and subsequent pro-inflammatory cytokine that results in progression to liver inflammation and hepatocyte injury in NASH (18, 66). It is reported that NLRP3 could also sense PGN fragments and transmit inflammatory signals (67). In this study, HGPP not only inhibited the transcriptional activity and expression of TLR2, NLRP3, TNF-α, and IL-1β genes but also induced the serum concentration of IL-1β in a murine MCD model of NASH. These results suggest that the effects of HGPP treatment might be mediated by inhibition of the TLR2/NLRP3 pathway through the modulation of the gut microbiota, but further studies are required to prove a mechanistic link.

In this study, we revealed the anti-inflammatory and hepatic protective effects of GPP and suggested that these may associate with the modulation of the gut microbiota and the TLR2/NLRP3 signaling pathway. A novel potential therapeutic role for GPP was characterized and provided a preliminary exploration of its underlying mechanism. This, to our knowledge, is the first study to examine the effects of GPP in NASH, suggesting that the mechanism might be preliminarily initiated by the modulation of the gut microbiota and TLR2/NLRP3 pathway. It is indicated that GPP could be used as a prebiotic agent for therapy of inflammation-related NASH patients and contribute to the potential gut microbiota involving mechanisms of natural herbs in NASH. Future studies should further explore certain bacteria and altered gut microbiota-related metabolites as biomarkers for disordered gut microbiota by GPP treatment.

## Data availability statement

The datasets presented in this study can be found in online repositories. The names of the repository/repositories and accession number(s) can be found below: https://www.ncbi.nlm.nih.gov/, SRP361247 https://www.ncbi.nlm.nih.gov/, GSE205794.

## Ethics Statement

All procedures performed in the study involving animals were in accordance with the ethical standards of and protocol approved by the Shanghai Model Organisms Center.

## Author contributions

S-RY and R-RW designed the experiments; S-RY and Y-YT performed the experiments; S-RY and Y-YT analyzed the data; S-RY wrote the original manuscript; LZ, GJ, and B-CL supervised the research; S-RY, B-JZ, and F-YJ collaborated in the characterization of GPP; LZ, GJ, B-CL, and R-RW reviewed and edited the manuscript critically. All authors have read and agreed to the published version of the manuscript.

## Funding

This work was supported by the National Natural Science Foundation of China (No. 82004149 and No. 81973730), Shanghai Collaborative Innovation Center for Chronic Disease Prevention and Health Services (2021 Science and Technology 02-37), Shanghai Science and Technology Planning Program (21DZ2271000), and Local Colleges Faculty Constitution of Shanghai MSTC (22010504300).

## Conflict of Interest

The authors declare that the research was conducted in the absence of any commercial or financial relationships that could be construed as a potential conflict of interest.

## Publisher’s note

All claims expressed in this article are solely those of the authors and do not necessarily represent those of their affiliated organizations, or those of the publisher, the editors and the reviewers. Any product that may be evaluated in this article, or claim that may be made by its manufacturer, is not guaranteed or endorsed by the publisher.

## Supplementary Material

Supplementary Figure 1 Body weight development among MCS, MCD, LGPP, HGPP, and PPC groups at week 0/1/2/3/4.

Supplementary Figure 2 FPKM values of GAPDH gene among MCS, MCD, and HGPP groups.

Supplementary Figure 3 Gene expression profiles among MCS, MCD, and HGPP groups.

Supplementary Figure 4 The western blotting analysis of Caspase-1/β-actin and Pro-caspase-1/β-actin.

Supplementary Table 1 Primer sequences for qPCR.

Supplementary Table 2 The RIN value, total amount and concentration of RNA, and amount for RNA sequencing.

Supplementary Tables 3–5 Top 20 of Gene ontology (GO) of biological processes of enrichment terms between MCS vs MCD group, MCD vs HGPP group, and MCS vs HGPP group (*P* value < 0.05).

Supplementary TableS 6–8 Top 20 of Kyoto Encyclopedia of Genes and Genomes (KEGG) enrichment pathways between MCS vs MCD group, MCD vs HGPP group, and MCS vs HGPP group (*P* value < 0.05).

Supplementary TableS 9–11 Gene Set Enrichment Analysis (GSEA) of KEGG pathway between MCS vs MCD group, MCD vs HGPP group, and MCS vs HGPP group (|NES|>1 and *P* adjust <0.05).

## References

[1] Byrne CD Targher G. NAFLD: A multisystem disease. J Hepatol (2015) 621 Suppl:S47–64. doi: 10.1016/j.jhep.2014.12.012 25920090

[2] Schuster S Cabrera D Arrese M Feldstein AE. Triggering and resolution of inflammation in NASH. Nat Rev Gastroenterol Hepatol (2018) 156:349–64. doi: 10.1038/s41575-018-0009-6 29740166

[3] Newsome PN Buchholtz K Cusi K Linder M Okanoue T Ratziu V. A placebo-controlled trial of subcutaneous semaglutide in nonalcoholic steatohepatitis. N Engl J Med (2021) 38412:1113–24. doi: 10.1056/NEJMoa2028395 33185364

[4] Younossi ZM Koenig AB Abdelatif D Fazel Y Henry L Wymer M. Global epidemiology of nonalcoholic fatty liver disease-Meta-Analytic assessment of prevalence, incidence, and outcomes. Hepatology (2016) 641:73–84. doi: 10.1002/hep.28431 26707365

[5] Koyama Y Brenner DA. Liver inflammation and fibrosis. J Clin Invest (2017) 1271:55–64. doi: 10.1172/JCI88881 PMC5199698 28045404

[6] Calzadilla Bertot L Adams LA. The natural course of non-alcoholic fatty liver disease. Int J Mol Sci (2016) 175:774. doi: 10.3390/ijms17050774 PMC4881593 27213358

[7] Zhang XJ She ZG HLL. Time to step-up the fight against NAFLD. Hepatology (2018) 676:2068–71. doi: 10.1002/hep.29845 29451316

[8] Brandl K Schnabl B. Intestinal microbiota and nonalcoholic steatohepatitis. Curr Opin Gastroenterol (2017) 333:128–33. doi: 10.1097/MOG.0000000000000349 PMC5662009 28257306

[9] Kaden-Volynets V Basic M Neumann U Pretz D Rings A Bleich A. Lack of liver steatosis in germ-free mice following hypercaloric diets. Eur J Nutr (2019) 585:1933–45. doi: 10.1007/s00394-018-1748-4 29926176

[10] Yuan J Chen C Cui J Lu J Yan C Wei X. Fatty liver disease caused by high-Alcohol-Producing klebsiella pneumoniae. Cell Metab (2019) 304:675–688.e677. doi: 10.1016/j.cmet.2019.08.018 31543403

[11] Aron-Wisnewsky J Vigliotti C Witjes J Le P Holleboom AG Verheij J. Gut microbiota and human NAFLD: Disentangling microbial signatures from metabolic disorders. Nat Rev Gastroenterol Hepatol (2020) 175:279–97. doi: 10.1038/s41575-020-0269-9 32152478

[12] Formes H Bernardes JP Mann A Bayer F Pontarollo G Kiouptsi K. The gut microbiota instructs the hepatic endothelial cell transcriptome. iScience (2021) 2410:103092. doi: 10.1016/j.isci.2021.103092 PMC8479694 34622147

[13] Rahman K Desai C Iyer SS Thorn NE Kumar P Liu Y. Loss of junctional adhesion molecule a promotes severe steatohepatitis in mice on a diet high in saturated fat, fructose, and cholesterol. Gastroenterology (2016) 1514:733–746.e712. doi: 10.1053/j.gastro.2016.06.022 PMC5037035 27342212

[14] Zhu L Baker RD Baker SS. Gut microbiome and nonalcoholic fatty liver diseases. Pediatr Res (2015) 771-2:245–51. doi: 10.1038/pr.2014.157 25310763

[15] Cani PD Amar J Iglesias MA Poggi M Knauf C Bastelica D. Metabolic endotoxemia initiates obesity and insulin resistance. Diabetes (2007) 567:1761–72. doi: 10.2337/db06-1491 17456850

[16] Csak T Velayudham A Hritz I Petrasek J Levin I Lippai D. Deficiency in myeloid differentiation factor-2 and toll-like receptor 4 expression attenuates nonalcoholic steatohepatitis and fibrosis in mice. Am J Physiol Gastrointest Liver Physiol (2011) 3003:G433–441. doi: 10.1152/ajpgi.00163.2009 PMC3302188 21233280

[17] Canfora EE Meex RCR Venema K Blaak EE. Gut microbial metabolites in obesity, NAFLD and T2DM. Nat Rev Endocrinol (2019) 155:261–73. doi: 10.1038/s41574-019-0156-z 30670819

[18] Miura K Yang L van Rooijen N Brenner DA Ohnishi H Seki E. Toll-like receptor 2 and palmitic acid cooperatively contribute to the development of nonalcoholic steatohepatitis through inflammasome activation in mice. Hepatology (2013) 572:577–89. doi: 10.1002/hep.26081 PMC3566276 22987396

[19] Shi KQ Fan YC Liu WY Li LF Chen YP Zheng MH. Traditional Chinese medicines benefit to nonalcoholic fatty liver disease: A systematic review and meta-analysis. Mol Biol Rep (2012) 3910:9715–22. doi: 10.1007/s11033-012-1836-0 22718512

[20] Chen MT Hou PF Zhou M Ren QB Wang XL Huang L. Resveratrol attenuates high-fat diet-induced non-alcoholic steatohepatitis by maintaining gut barrier integrity and inhibiting gut inflammation through regulation of the endocannabinoid system. Clin Nutr (2020) 394:1264–75. doi: 10.1016/j.clnu.2019.05.020 31189495

[21] Chen YY Zhao ZM Fan HN Li ZX He YC Liu CH. Safety and therapeutic effects of anti-fibrotic traditional Chinese medicine fuzheng huayu on persistent advanced stage fibrosis following 2 years entecavir treatment: Study protocol for a single arm clinical objective performance criteria trial. Contemp Clin Trials Commun (2020) 19:100601. doi: 10.1016/j.conctc.2020.100601 32642592 PMC7334581

[22] Hu L Chen Z Xie Y. New triterpenoid saponins from gynostemma pentaphyllum. J Nat Prod (1996) 5912:1143–5. doi: 10.1021/np960445u 8988599

[23] Jia D Rao CG Xue SX Lei JL. Purification, characterization and neuroprotective effects of a polysaccharide from gynostemma pentaphyllum. Carbohydr Polym (2015) 122:93–100. doi: 10.1016/j.carbpol.2014.12.032 25817647

[24] Shang QS Song GR Zhang MF Shi JJ Xu CY. Dietary fucoidan improves metabolic syndrome in association with increased akkermansia population in the gut microbiota of high-fat diet-fed mice - ScienceDirect. J Funct Foods (2017) 28:138–46. doi: 10.1016/j.jff.2016.11.002

[25] Zhu LY Li J Wei CH Luo T Deng ZY Fan YW. A polysaccharide from fagopyrum esculentum moench bee pollen alleviates microbiota dysbiosis to improve intestinal barrier function in antibiotic-treated mice. Food Funct (2020) 1112:10519–33. doi: 10.1039/d0fo01948h 33179663

[26] Gao W Feng Z Zhang S Wu B Peng C. Anti-inflammatory and antioxidant effect of eucommia ulmoides polysaccharide in hepatic ischemia-reperfusion injury by regulating ROS and the TLR-4-NF- κ b pathway. BioMed Res Int (2020) 2020:1–11. doi: 10.1155/2020/1860637 PMC7273391 32566664

[27] Wu TR Lin CS Chang CJ Lin TL Martel J Ko YF. Gut commensal parabacteroides goldsteinii plays a predominant role in the anti-obesity effects of polysaccharides isolated from hirsutella sinensis. Gut (2019) 682:248–62. doi: 10.1136/gutjnl-2017-315458 30007918

[28] De Vadder F Kovatcheva-Datchary P Goncalves D Vinera J Zitoun C Duchampt A. Microbiota-generated metabolites promote metabolic benefits via gut-brain neural circuits. Cell (2014) 1561-2:84–96. doi: 10.1016/j.cell.2013.12.016 24412651

[29] Nakamura YK Omaye ST. Metabolic diseases and pro- and prebiotics: Mechanistic insights. Nutr Metab (Lond) (2012) 91:60. doi: 10.1186/1743-7075-9-60 PMC3464869 22713169

[30] Parnell JA Reimer RA. Prebiotic fiber modulation of the gut microbiota improves risk factors for obesity and the metabolic syndrome. Gut Microbes (2012) 31:29–34. doi: 10.4161/gmic.19246 PMC3827018 22555633

[31] Kleiner DE Brunt EM Van Natta M Behling C Contos MJ Cummings OW. Design and validation of a histological scoring system for nonalcoholic fatty liver disease. Hepatology (2005) 416:1313–21. doi: 10.1002/hep.20701 15915461

[32] Tang Q Lim T Wei XJ Wang QY Xu JC Shen LY. A free-standing multilayer film as a novel delivery carrier of platelet lysates for potential wound-dressing applications. Biomaterials (2020) 255:120138. doi: 10.1016/j.biomaterials.2020.120138 32521330

[33] Nemet I Saha PP Gupta N Zhu W Romano KA Skye SM. A cardiovascular disease-linked gut microbial metabolite acts *via* adrenergic receptors. Cell (2020) 1805:862–877.e822. doi: 10.1016/j.cell.2020.02.016 PMC7402401 32142679

[34] Askarian F Wagner T Johannessen M Nizet V. Staphylococcus aureus modulation of innate immune responses through toll-like (TLR), (NOD)-like (NLR) and c-type lectin (CLR) receptors. FEMS Microbiol Rev (2018) 425:656–71. doi: 10.1093/femsre/fuy025 PMC6098222 29893825

[35] Kumar S Ingle H Prasad DV Kumar H. Recognition of bacterial infection by innate immune sensors. Crit Rev Microbiol (2013) 393:229–46. doi: 10.3109/1040841x.2012.706249 22866947

[36] Allen IC Wilson JE Schneider M Lich JD Roberts RA Arthur JC. NLRP12 suppresses colon inflammation and tumorigenesis through the negative regulation of noncanonical NF-κb signaling. Immunity (2012) 365:742–54. doi: 10.1016/j.immuni.2012.03.012 PMC3658309 22503542

[37] Yang J Zhao Y Shi J Shao F. Human NAIP and mouse NAIP1 recognize bacterial type III secretion needle protein for inflammasome activation. Proc Natl Acad Sci U.S.A. (2013) 11035:14408–13. doi: 10.1073/pnas.1306376110 PMC3761597 23940371

[38] Chou W-C Guo Z Guo H Chen L Zhang G Liang K. AIM2 in regulatory T cells restrains autoimmune diseases. Nature (2021) 5917849:300–5. doi: 10.1038/s41586-021-03231-w PMC8080937 33505023

[39] Lamas B Richard ML Leducq V Pham HP Michel ML Da Costa G. CARD9 impacts colitis by altering gut microbiota metabolism of tryptophan into aryl hydrocarbon receptor ligands. Nat Med (2016) 226:598–605. doi: 10.1038/nm.4102 PMC5087285 27158904

[40] Vonderheide RH. The immune revolution: A case for priming, not checkpoint. Cancer Cell (2018) 334:563–9. doi: 10.1016/j.ccell.2018.03.008 PMC5898647 29634944

[41] Collins M Ling V Carreno BM. The B7 family of immune-regulatory ligands. Genome Biol (2005) 66:223. doi: 10.1186/gb-2005-6-6-223 PMC1175965 15960813

[42] Estes C Razavi H Loomba R Younossi Z Sanyal AJ. Modeling the epidemic of nonalcoholic fatty liver disease demonstrates an exponential increase in burden of disease. Hepatology (2018) 671:123–33. doi: 10.1002/hep.29466 PMC5767767 28802062

[43] Yan TT Yan NN Wang P Xia YL Hao HP Wang GJ. Herbal drug discovery for the treatment of nonalcoholic fatty liver disease. Acta Pharm Sin B (2020) 101:3–18. doi: 10.1016/j.apsb.2019.11.017 PMC6977016 31993304

[44] Liu HL Lv J Zhao ZM Xiong AM Tan Y Glenn JS. Fuzhenghuayu decoction ameliorates hepatic fibrosis by attenuating experimental sinusoidal capillarization and liver angiogenesis. Sci Rep (2019) 91:18719. doi: 10.1038/s41598-019-54663-4 PMC6904731 31822697

[45] Li CL Zhou WJ Li M Shu XB Zhang L Ji G. Salvia-nelumbinis naturalis extract protects mice against MCD diet-induced steatohepatitis *via* activation of colonic FXR-FGF15 pathway. BioMed Pharmacother (2021) 139:111587. doi: 10.1016/j.biopha.2021.111587 33865013

[46] Lee JH Oh JY Kim SH Oh IJ Lee YH Lee KW. Pharmaceutical efficacy of gypenoside LXXV on non-alcoholic steatohepatitis (NASH). Biomolecules (2020) 1010:1426. doi: 10.3390/biom10101426 PMC7599508 33050067

[47] Xia T Liu CS Hu YN Luo ZY Chen FL Yuan LX. Coix seed polysaccharides alleviate type 2 diabetes mellitus *via* gut microbiota-derived short-chain fatty acids activation of IGF1/PI3K/AKT signaling. Food Res Int (2021) 150Pt A:110717. doi: 10.1016/j.foodres.2021.110717 34865748

[48] Wang C Yin Y Cao X Li X. Effects of maydis stigma polysaccharide on the intestinal microflora in type-2 diabetes. Pharm Biol (2016) 5412:3086–92. doi: 10.1080/13880209.2016.1211153 27558859

[49] Zhu KX Nie SP Tan LH Li C Gong DM Xie MY. A polysaccharide from ganoderma atrum improves liver function in type 2 diabetic rats *via* antioxidant action and short-chain fatty acids excretion. J Agric Food Chem (2016) 649:1938–44. doi: 10.1021/acs.jafc.5b06103 26898215

[50] Huang J Liu D Wang Y Liu L Li J Yuan J. Ginseng polysaccharides alter the gut microbiota and Kynurenine/Tryptophan ratio, potentiating the antitumour effect of antiprogrammed cell death 1/Programmed cell death ligand 1 (Anti-PD-1/PD-L1) immunotherapy. Gut (2022) 714:734–45. doi: 10.1136/gutjnl-2020-321031 PMC8921579 34006584

[51] Lin TL Lu CC Lai WF Wu TS Lu JJ Chen YM. Role of gut microbiota in identification of novel TCM-derived active metabolites. Protein Cell (2021) 125:394–410. doi: 10.1007/s13238-020-00784-w PMC8106560 32929698

[52] Schneeberger M Everard A Gomez-Valades AG Matamoros S Ramirez S Delzenne NM. Akkermansia muciniphila inversely correlates with the onset of inflammation, altered adipose tissue metabolism and metabolic disorders during obesity in mice. Sci Rep (2015) 5:16643. doi: 10.1038/srep16643 26563823 PMC4643218

[53] Wu WR Lv LX Shi D Ye JZ Fang DQ Guo FF. Protective effect of akkermansia muciniphila against immune-mediated liver injury in a mouse model. Front Microbiol (2017) 8:1804. doi: 10.3389/fmicb.2017.01804 29033903 PMC5626943

[54] Kelly JR Kennedy PJ Cryan JF Dinan TG Clarke G Hyland NP. Breaking down the barriers: The gut microbiome, intestinal permeability and stress-related psychiatric disorders. Front Cell Neurosci (2015) 9:392. doi: 10.3389/fncel.2015.00392 26528128 PMC4604320

[55] Pan FW Zhang LY Li M Hu YX Zeng BH Yuan HJ. Predominant gut lactobacillus murinus strain mediates anti-inflammaging effects in calorie-restricted mice. Microbiome (2018) 61:54. doi: 10.1186/s40168-018-0440-5 PMC5863386 29562943

[56] Xu Y Wang N Tan HY Li S Zhang C Feng YB. Function of akkermansia muciniphila in obesity: Interactions with lipid metabolism, immune response and gut systems. Front Microbiol (2020) 11:219. doi: 10.3389/fmicb.2020.00219 32153527 PMC7046546

[57] Hoyles L Fernandez-Real JM Federici M Serino M Abbott J Charpentier J. Molecular phenomics and metagenomics of hepatic steatosis in non-diabetic obese women. Nat Med (2018) 247:1070–80. doi: 10.1038/s41591-018-0061-3 PMC6140997 29942096

[58] Everard A Lazarevic V Gaia N Johansson M Stahlman M Backhed F. Microbiome of prebiotic-treated mice reveals novel targets involved in host response during obesity. ISME J (2014) 810:2116–30. doi: 10.1038/ismej.2014.45 PMC4163056 24694712

[59] Kolodziejczyk AA Zheng D Shibolet O Elinav E. The role of the microbiome in NAFLD and NASH. EMBO Mol Med (2019) 112:e9302. doi: 10.15252/emmm.201809302 PMC6365925 30591521

[60] Cario E Gerken G Podolsky DK. Toll-like receptor 2 controls mucosal inflammation by regulating epithelial barrier function. Gastroenterology (2007) 1324:1359–74. doi: 10.1053/j.gastro.2007.02.056 17408640

[61] Lee J Park S Oh N Park J Kwon M Seo J. Oral intake of lactobacillus plantarum l-14 extract alleviates TLR2- and AMPK-mediated obesity-associated disorders in high-Fat-Diet-Induced obese C57BL/6J mice. Cell Prolif (2021) 546:e13039. doi: 10.1111/cpr.13039 PMC8168423 33830560

[62] Jäckel S Kiouptsi K Lillich M Hendrikx T Khandagale A Kollar B. Gut microbiota regulate hepatic Von willebrand factor synthesis and arterial thrombus formation *via* toll-like receptor-2. Blood (2017) 1304:542–53. doi: 10.1182/blood-2016-11-754416 28572286

[63] Wolf AJ Underhill DM. Peptidoglycan recognition by the innate immune system. Nat Rev Immunol (2018) 184:243–54. doi: 10.1038/nri.2017.136 29292393

[64] King JE Roberts IS. Bacterial surfaces: Front lines in host-pathogen interaction. Adv Exp Med Biol (2016) 915:129–56. doi: 10.1007/978-3-319-32189-9_10 27193542

[65] Jiang D Chen S Sun R Zhang X Wang D. The NLRP3 inflammasome: Role in metabolic disorders and regulation by metabolic pathways. Cancer Lett (2018) 419:8–19. doi: 10.1016/j.canlet.2018.01.034 29339210

[66] Mridha AR Wree A Robertson AAB Yeh MM Johnson CD Van Rooyen DM. NLRP3 inflammasome blockade reduces liver inflammation and fibrosis in experimental NASH in mice. J Hepatol (2017) 665:1037–46. doi: 10.1016/j.jhep.2017.01.022 PMC6536116 28167322

[67] Wolf AJ Reyes CN Liang W Becker C Shimada K Wheeler ML. Hexokinase is an innate immune receptor for the detection of bacterial peptidoglycan. Cell (2016) 1663:624–36. doi: 10.1016/j.cell.2016.05.076 PMC5534359 27374331

