# Supplementary material for: Gynostemma pentaphyllum polysaccharides ameliorate non-alcoholic steatohepatitis in mice associated with gut microbiota and the TLR2/NLRP3 pathway
Source: Front Endocrinol (Lausanne). 2022 Jul 22;13:885039. doi: 10.3389/fendo.2022.885039 (PMC9352886; doi:10.3389/fendo.2022.885039)
Supplement: Supplementary Figure 1 — Body weight development among MCS, MCD, LGPP, HGPP, and PPC groups at week 0/1/2/3/4. [file DataSheet_1.docx]

Supplementary Material


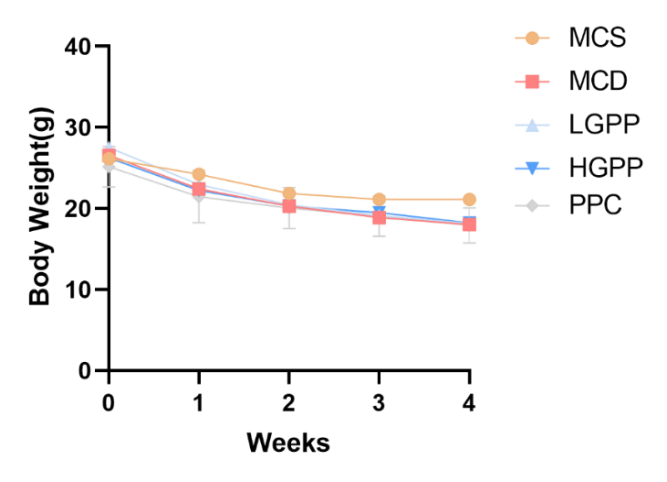


**Supplementary Figure 1.** Body weight development among MCS, MCD, LGPP, HGPP, and PPC groups at week 0/1/2/3/4 (n=7).

**
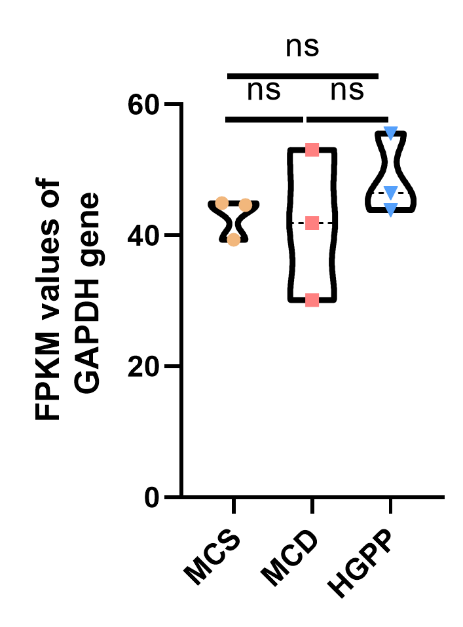
**

**Supplementary Figure 2.** FPKM values of GAPDH gene among MCS, MCD, and HGPP groups. (n=3).

| Gene | Forward | Reverse |
| --- | --- | --- |
| TLR2 | AAGATGCGCTTCCTGAATTTG | TCCAGCGTCTGAGGAATGC |
| NLRP3 | TCCACAATTCTGACCCACAA | ACCTCACAGAGGGTCACCAC |
| ASC | CTTGTCAGGGGATGAACTCAAAA | GCCATACGACTCCAGATAGTAGC |
| Caspase-1 | ACAAGGCACGGGACCTATG | TCCCAGTCAGTCCTGGAAATG |
| IL-1β | GAAATGCCACCTTTTGACAGTG | CTGGATGCTCTCATCAGGACA |
| TNF-α | ACTCCAGGCGGTGCCTATGT | AGTGTGAGGGTCTGGGCCAT |
| GAPDH | TGTTTCCTCGTCCCGTAG | TGTTTCCTCGTCCCGTAG |

**Supplementary Table 1.** Primer sequences for qPCR

| Number | RIN value | Total amount of RNA(μg) | Concentration(μg/ml) | Amount |
| --- | --- | --- | --- | --- |
| MCS1 | 7.8 | 21.006 | 381.92 | 3 |
| MCS2 | 7.8 | 33.492 | 446.56 |  |
| MCS3 | 8 | 19.777 | 359.59 |  |
| MCD1 | 7.5 | 13.286 | 379.59 | 3 |
| MCD2 | 7.8 | 21.131 | 384.19 |  |
| MCD3 | 7.3 | 19.812 | 360.22 |  |
| HGPP1 | 8 | 18.167 | 330.31 | 3 |
| HGPP2 | 7.7 | 13.172 | 376.34 |  |
| HGPP3 | 8.7 | 20.163 | 366.6 |  |

**Supplementary Table 2.** The RIN value, total amount and concentration of RNA, and amount for RNA sequencing.


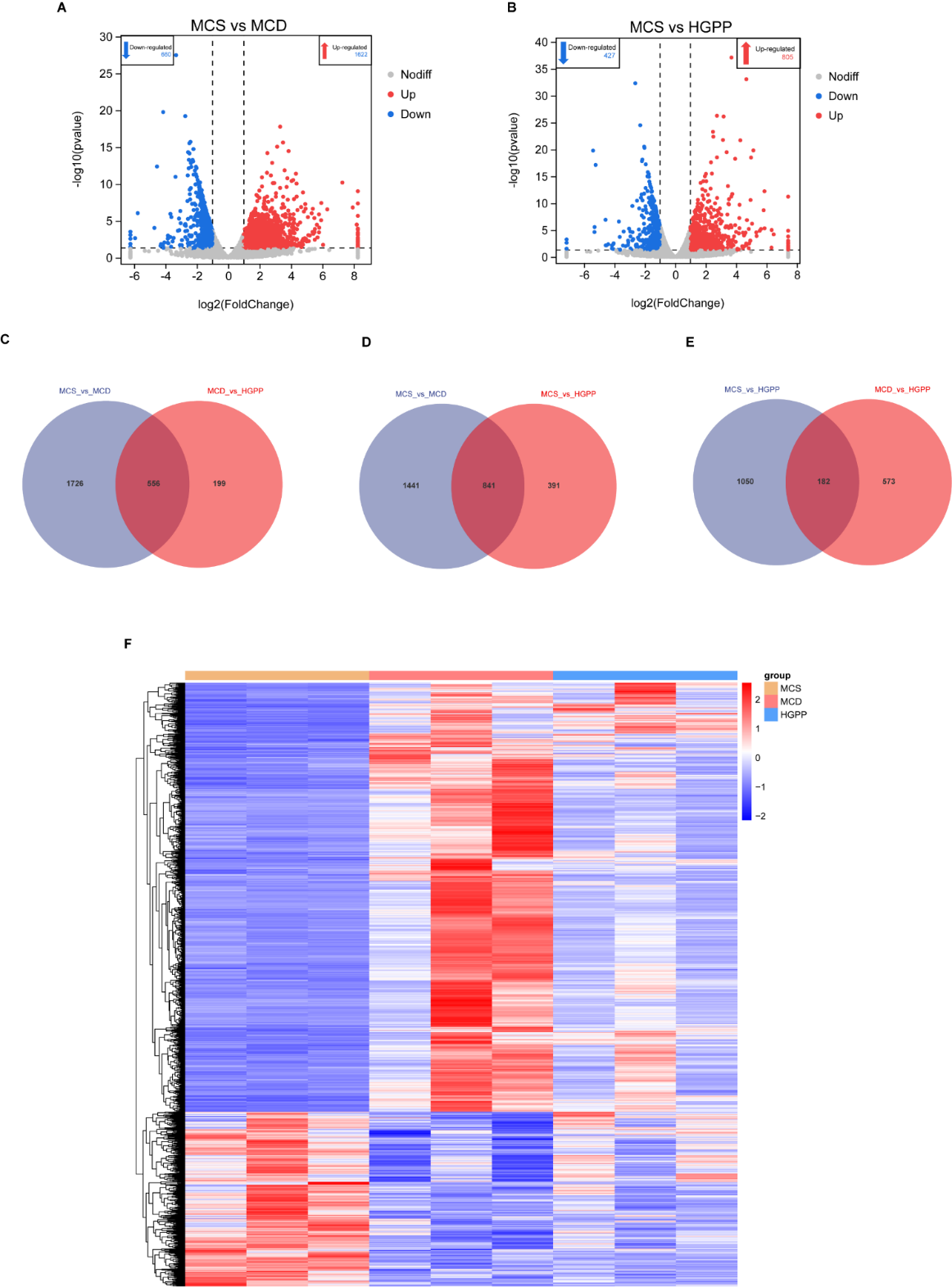


**Supplementary Figure 3.** Gene expression profiles among MCS, MCD, and HGPP groups (n=3). **(A)** Screening different genes on MCS vs MCD by Volcano plot (|log_2_Foldchange|>1, *P* value<0.05); **(B)** Screening different genes on MCS vs HGPP by Volcano plot (|log_2_Foldchange|>1, *P* value<0.05); **(C)** Venn diagram showing the DEGs profiling at MCS vs MCD, and MCD vs HGPP, with each section showing the gene expression number; **(D)** Venn diagram showing the differentially expressed genes (DEGs) comparing the three groups DEGs profiling at MCS vs MCD, and MCS vs HGPP, with each section showing the gene expression number; **(E)** Venn diagram showing the DEGs profiling at MCS vs HGPP, and MCD vs HGPP, with each section showing the gene expression number; **(F)** Heatmap for DEGs between the MCS, MCD, and HGPP groups.

| GO enrichment term | DEG | *P* value | *P* adjust |
| --- | --- | --- | --- |
| Immune system process | 525 | 5.67E-66 | 5.67E-62 |
| Immune response | 357 | 3.90E-60 | 1.95E-56 |
| Response to external stimulus | 541 | 1.22E-53 | 4.05E-50 |
| Defense response | 353 | 6.97E-49 | 1.74E-45 |
| Regulation of immune system process | 301 | 2.89E-43 | 5.79E-40 |
| Response to other organism | 317 | 3.93E-43 | 6.56E-40 |
| Cellular response to chemical stimulus | 552 | 5.15E-43 | 7.36E-40 |
| Response to external biotic stimulus | 317 | 6.32E-43 | 7.90E-40 |
| Response to stress | 633 | 1.38E-42 | 1.54E-39 |
| Response to biotic stimulus | 319 | 1.22E-41 | 1.22E-38 |
| Inflammatory response | 201 | 2.84E-41 | 2.58E-38 |
| Cell activation | 243 | 7.84E-41 | 6.54E-38 |
| Positive regulation of immune system process | 221 | 5.28E-39 | 4.07E-36 |
| Regulation of response to stimulus | 631 | 6.35E-39 | 4.54E-36 |
| Innate immune response | 197 | 1.49E-37 | 9.96E-35 |
| Leukocyte activation | 217 | 2.06E-37 | 1.29E-34 |
| Small molecule metabolic process | 357 | 7.86E-37 | 4.63E-34 |
| Interspecies interaction between organisms | 333 | 1.22E-36 | 6.80E-34 |
| Defense response to other organism | 241 | 4.94E-36 | 2.60E-33 |
| Regulation of response to external stimulus | 218 | 1.45E-33 | 7.24E-31 |

**Supplementary Table 3.** Top 20 of Gene ontology (GO) of biological processes of enrichment terms between MCS vs MCD group (*P* value < 0.05).

| GO enrichment term | DEG | *P* value | *P* adjust |
| --- | --- | --- | --- |
| Immune system process | 228 | 1.41E-48 | 9.41E-45 |
| Leukocyte activation | 125 | 2.98E-45 | 9.96E-42 |
| Cell activation | 133 | 5.60E-45 | 1.25E-41 |
| Immune response | 155 | 1.62E-39 | 2.72E-36 |
| Regulation of immune system process | 144 | 1.00E-37 | 1.34E-34 |
| Lymphocyte activation | 102 | 1.33E-36 | 1.48E-33 |
| Positive regulation of immune system process | 108 | 7.40E-33 | 7.07E-30 |
| Response to external stimulus | 205 | 1.92E-28 | 1.61E-25 |
| Leukocyte migration | 64 | 1.12E-27 | 7.84E-25 |
| Regulation of cell activation | 80 | 1.17E-27 | 7.84E-25 |
| Inflammatory response | 90 | 2.82E-27 | 1.72E-24 |
| T cell activation | 74 | 3.57E-27 | 1.99E-24 |
| Defense response | 140 | 5.17E-27 | 2.51E-24 |
| Regulation of leukocyte activation | 76 | 5.26E-27 | 2.51E-24 |
| Cellular response to chemical stimulus | 211 | 4.77E-25 | 2.13E-22 |
| Cytokine production | 90 | 8.89E-25 | 3.72E-22 |
| Response to other organism | 127 | 1.32E-24 | 5.21E-22 |
| Response to external biotic stimulus | 127 | 1.61E-24 | 5.98E-22 |
| Positive regulation of cytokine production | 67 | 1.76E-24 | 6.18E-22 |
| Regulation of immune response | 82 | 1.92E-24 | 6.43E-22 |

**Supplementary Table 4.** Top 20 of Gene ontology (GO) of biological processes of enrichment terms between MCD vs HGPP group (*P* value < 0.05).

| GO enrichment term | DEG | *P* value | *P* adjust |
| --- | --- | --- | --- |
| Small molecule metabolic process | 222 | 1.28E-31 | 1.02E-27 |
| Organic acid metabolic process | 145 | 3.47E-26 | 1.38E-22 |
| Carboxylic acid metabolic process | 134 | 6.09E-24 | 1.61E-20 |
| Oxoacid metabolic process | 136 | 4.39E-23 | 8.72E-20 |
| Cellular response to chemical stimulus | 296 | 1.01E-22 | 1.60E-19 |
| Response to stress | 336 | 1.52E-21 | 2.01E-18 |
| Response to external stimulus | 269 | 6.84E-21 | 7.76E-18 |
| Monocarboxylic acid metabolic process | 97 | 2.80E-20 | 2.78E-17 |
| Wound healing | 76 | 2.15E-19 | 1.89E-16 |
| Response to wounding | 87 | 2.06E-18 | 1.63E-15 |
| Phosphorus metabolic process | 283 | 1.19E-17 | 8.14E-15 |
| Lipid metabolic process | 153 | 1.23E-17 | 8.14E-15 |
| Phosphate-containing compound metabolic process | 280 | 3.08E-17 | 1.88E-14 |
| Oxidation-reduction process | 132 | 3.83E-17 | 2.17E-14 |
| Drug metabolic process | 85 | 6.77E-17 | 3.59E-14 |
| Response to organic substance | 294 | 3.08E-16 | 1.53E-13 |
| Regulation of response to stimulus | 321 | 4.74E-16 | 2.21E-13 |
| Response to oxygen-containing compound | 182 | 8.46E-16 | 3.73E-13 |
| Regulation of biological quality | 330 | 1.32E-15 | 5.51E-13 |
| Defense response | 161 | 7.24E-15 | 2.87E-12 |

**Supplementary Table 5.** Top 20 of Gene ontology (GO) of biological processes of enrichment terms between MCS vs HGPP group (*P* value < 0.05).

| KEGG pathway | DEG | *P* value | *P* adjust |
| --- | --- | --- | --- |
| Complement and coagulation cascades | 36 | 1.01E-10 | 3.13E-08 |
| Chemokine signaling pathway | 52 | 2.46E-07 | 9.49E-06 |
| Osteoclast differentiation | 37 | 5.76E-07 | 1.74E-05 |
| Hematopoietic cell lineage | 29 | 5.46E-06 | 0.00013 |
| Nod-like receptor signaling pathway | 43 | 7.19E-06 | 0.000131 |
| C-type lectin receptor signaling pathway | 32 | 8.97E-06 | 0.000152 |
| Fc gamma R-mediated phagocytosis | 24 | 5.76E-05 | 0.000712 |
| Platelet activation | 32 | 7.12E-05 | 0.000846 |
| Intestinal immune network for IgA production | 15 | 0.000167 | 0.001669 |
| Inflammatory mediator regulation of TRP channels | 31 | 0.000237 | 0.002156 |
| B cell receptor signaling pathway | 20 | 0.000389 | 0.003083 |
| Bile secretion | 22 | 0.000459 | 0.003459 |
| Natural killer cell mediated cytotoxicity | 30 | 0.001813 | 0.010622 |
| Toll-like receptor signaling pathway | 24 | 0.001822 | 0.010622 |
| Thyroid hormone synthesis | 19 | 0.001868 | 0.010689 |
| Fc epsilon RI signaling pathway | 17 | 0.004169 | 0.020776 |
| Leukocyte transendothelial migration | 25 | 0.006917 | 0.031432 |
| Carbohydrate digestion and absorption | 12 | 0.007684 | 0.033918 |
| Progesterone-mediated oocyte maturation | 20 | 0.011592 | 0.047759 |
| Estrogen signaling pathway | 27 | 0.012982 | 0.052781 |

**Supplementary Table 6.** Top 20 of Kyoto Encyclopedia of Genes and Genomes (KEGG) enrichment pathways between MCS vs MCD group (*P* value < 0.05).

| KEGG enrichment pathway | DEG | *P* value | *P* adjust |
| --- | --- | --- | --- |
| Chemokine signaling pathway | 32 | 1.57E-10 | 4.32E-08 |
| Osteoclast differentiation | 24 | 9.27E-10 | 1.28E-07 |
| Hematopoietic cell lineage | 17 | 8.61E-07 | 3.95E-05 |
| Natural killer cell mediated cytotoxicity | 20 | 2.00E-06 | 6.87E-05 |
| Fc gamma R-mediated phagocytosis | 13 | 5.53E-05 | 0.001268 |
| Intestinal immune network for IgA production | 9 | 8.69E-05 | 0.001838 |
| Platelet activation | 16 | 0.000117 | 0.002295 |
| Leukocyte transendothelial migration | 15 | 0.000202 | 0.003705 |
| C-type lectin receptor signaling pathway | 14 | 0.000465 | 0.006879 |
| Toll-like receptor signaling pathway | 13 | 0.000498 | 0.006879 |
| Nod-like receptor signaling pathway | 18 | 0.000544 | 0.007121 |
| Fc epsilon RI signaling pathway | 10 | 0.00071 | 0.008429 |
| B cell receptor signaling pathway | 10 | 0.001014 | 0.010721 |
| Inflammatory mediator regulation of TRP channels | 14 | 0.001411 | 0.012521 |
| IL-17 signaling pathway | 11 | 0.002187 | 0.016705 |
| Circadian entrainment | 11 | 0.004314 | 0.028249 |
| Cholinergic synapse | 12 | 0.004672 | 0.029881 |
| Glutamatergic synapse | 11 | 0.010974 | 0.065607 |
| T cell receptor signaling pathway | 10 | 0.014952 | 0.085661 |
| Antigen processing and presentation | 8 | 0.024482 | 0.132012 |

**Supplementary Table 7.** Top 20 of Kyoto Encyclopedia of Genes and Genomes (KEGG) enrichment pathways between MCD vs HGPP group (*P* value < 0.05).

| KEGG enrichment pathway | DEG | *P* value | *P* adjust |
| --- | --- | --- | --- |
| Retinol metabolism | 29 | 3.54E-12 | 5.11E-10 |
| Complement and coagulation cascades | 26 | 1.84E-10 | 1.75E-08 |
| Steroid hormone biosynthesis | 26 | 2.42E-10 | 1.75E-08 |
| Metabolism of xenobiotics by cytochrome P450 | 22 | 7.38E-10 | 4.27E-08 |
| Arachidonic acid metabolism | 22 | 1.07E-07 | 5.14E-06 |
| Drug metabolism - other enzymes | 21 | 4.85E-07 | 1.76E-05 |
| Drug metabolism - cytochrome P450 | 18 | 4.87E-07 | 1.76E-05 |
| Linoleic acid metabolism | 13 | 2.22E-05 | 0.000713 |
| Phenylalanine metabolism | 8 | 7.50E-05 | 0.002168 |
| ABC transporters | 12 | 0.000107 | 0.002729 |
| Bile secretion | 16 | 0.000113 | 0.002729 |
| Glutathione metabolism | 14 | 0.000156 | 0.003472 |
| Inflammatory mediator regulation of TRP channels | 20 | 0.000372 | 0.00716 |
| Pentose and glucuronate interconversions | 9 | 0.00042 | 0.007581 |
| Amino sugar and nucleotide sugar metabolism | 11 | 0.000564 | 0.009584 |
| Cell cycle | 19 | 0.000806 | 0.012945 |
| Osteoclast differentiation | 19 | 0.000893 | 0.013579 |
| Galactose metabolism | 8 | 0.001319 | 0.019054 |
| Ascorbate and aldarate metabolism | 7 | 0.002101 | 0.027474 |
| Glycolysis / Gluconeogenesis | 12 | 0.002187 | 0.027474 |

**Supplementary Table 8.** Top 20 of Kyoto Encyclopedia of Genes and Genomes (KEGG) enrichment pathways between MCS vs HGPP group (*P* value < 0.05).

| KEGG pathway | Size | ES | NES | *P* value | *P* adjust |
| --- | --- | --- | --- | --- | --- |
| Cytokine cytokine receptor interaction | 93 | -0.50281 | -1.71761 | 0.000103 | 0.003031 |
| Cell adhesion molecules cams | 76 | -0.53389 | -1.79238 | 0.000104 | 0.003031 |
| Leukocyte transendothelial migration | 68 | -0.54185 | -1.80179 | 0.000104 | 0.003031 |
| Natural killer cell mediated cytotoxicity | 60 | -0.55802 | -1.83237 | 0.000105 | 0.003031 |
| Hematopoietic cell lineage | 42 | -0.6386 | -2.00308 | 0.000109 | 0.003031 |
| Primary immunodeficiency | 20 | -0.73498 | -2.02977 | 0.00012 | 0.003031 |
| Toll-like receptor signaling pathway | 53 | -0.55742 | -1.80274 | 0.000213 | 0.004601 |
| Primary bile acid biosynthesis | 11 | 0.798061 | 2.497916 | 0.000412 | 0.007439 |
| Leishmania infection | 37 | -0.60725 | -1.86814 | 0.000443 | 0.007439 |
| Drug metabolism other enzymes | 21 | 0.627663 | 2.407274 | 0.00061 | 0.008015 |
| Dilated cardiomyopathy | 53 | -0.54299 | -1.75606 | 0.00064 | 0.008015 |
| Butanoate metabolism | 25 | 0.709124 | 2.829118 | 0.00073 | 0.008015 |
| Fatty acid metabolism | 26 | 0.560906 | 2.260436 | 0.000746 | 0.008015 |
| Propanoate metabolism | 27 | 0.57141 | 2.317518 | 0.000775 | 0.008015 |
| Tryptophan metabolism | 28 | 0.572616 | 2.356241 | 0.000796 | 0.008015 |
| Valine leucine and isoleucine degradation | 32 | 0.645335 | 2.801723 | 0.000887 | 0.008374 |
| Beta alanine metabolism | 17 | 0.588348 | 2.092245 | 0.001065 | 0.009361 |
| T cell receptor signaling pathway | 64 | -0.51442 | -1.70001 | 0.001153 | 0.009361 |
| Glycine serine and threonine metabolism | 20 | 0.57057 | 2.159806 | 0.001178 | 0.009361 |
| B cell receptor signaling pathway | 57 | -0.51698 | -1.6881 | 0.00127 | 0.009585 |
| Drug metabolism cytochrome p450 | 27 | 0.478251 | 1.939685 | 0.00155 | 0.0108 |
| Pantothenate and coa biosynthesis | 10 | 0.694287 | 2.095622 | 0.001574 | 0.0108 |
| Citrate cycle tca cycle | 12 | 0.667332 | 2.141408 | 0.001737 | 0.011275 |
| Peroxisome | 56 | 0.445513 | 2.188083 | 0.001792 | 0.011275 |
| Hypertrophic cardiomyopathy hcm | 46 | -0.5391 | -1.71029 | 0.001947 | 0.011757 |
| Steroid hormone biosynthesis | 14 | 0.638058 | 2.15507 | 0.002387 | 0.013861 |
| Fc epsilon ri signaling pathway | 44 | -0.5247 | -1.65597 | 0.00326 | 0.018231 |
| Fc gamma r mediated phagocytosis | 61 | -0.49019 | -1.61323 | 0.003686 | 0.019877 |
| Cell cycle | 82 | -0.45728 | -1.54471 | 0.00403 | 0.020985 |
| Porphyrin and chlorophyll metabolism | 17 | 0.573417 | 2.03915 | 0.00426 | 0.021441 |
| Calcium signaling pathway | 89 | -0.44905 | -1.5276 | 0.004622 | 0.022511 |
| Spliceosome | 57 | 0.333132 | 1.63873 | 0.005455 | 0.025502 |
| Lysine degradation | 28 | 0.43616266 | 1.79475376 | 0.00557325 | 0.02550183 |
| Arachidonic acid metabolism | 26 | -0.5729482 | -1.6640369 | 0.00658047 | 0.02922501 |
| Ecm receptor interaction | 56 | -0.4822676 | -1.5714886 | 0.00762389 | 0.03289163 |
| Regulation of autophagy | 12 | 0.58088382 | 1.86400402 | 0.00825011 | 0.03460462 |
| Nod-like receptor signaling pathway | 28 | -0.5496473 | -1.6177649 | 0.01040476 | 0.04246265 |
| Pyruvate metabolism | 22 | 0.45557468 | 1.76216502 | 0.01096067 | 0.04355424 |
| Starch and sucrose metabolism | 22 | 0.44950075 | 1.73867104 | 0.01225016 | 0.04743011 |
| Biosynthesis of unsaturated fatty acids | 11 | 0.57627557 | 1.8037329 | 0.01276245 | 0.04817826 |

**Supplementary Table 9.** Gene Set Enrichment Analysis (GSEA) of KEGG pathway between MCS vs MCD group. (|NES|＞1 and *P* adjust＜0.05)

| KEGG pathway | Size | ES | NES | *P* value | *P* adjust |
| --- | --- | --- | --- | --- | --- |
| Cytokine receptor interaction | 140 | -0.6395 | -1.97964 | 0.000101 | 0.00611 |
| Chemokine signaling pathway | 115 | -0.5765 | -1.77003 | 0.000101 | 0.00611 |
| Hematopoietic cell lineage | 47 | -0.71808 | -2.03125 | 0.000108 | 0.00611 |
| Natural killer cell mediated cytotoxicity | 70 | -0.59211 | -1.75264 | 0.000208 | 0.007866 |
| Primary immunodeficiency | 21 | -0.73229 | -1.84165 | 0.000238 | 0.007866 |
| Neuroactive ligand receptor interaction | 88 | -0.54881 | -1.65668 | 0.000307 | 0.007866 |
| Leishmania infection | 38 | -0.66472 | -1.83118 | 0.00033 | 0.007866 |
| Intestinal immune network for IGA production | 17 | -0.78109 | -1.88863 | 0.00037 | 0.007866 |
| Calcium signaling pathway | 102 | -0.52585 | -1.60434 | 0.000508 | 0.009204 |
| Toll-like receptor signaling pathway | 61 | -0.58355 | -1.70302 | 0.000629 | 0.009204 |
| Citrate cycle (TCA cycle) | 22 | 0.651188 | 2.26834 | 0.000645 | 0.009204 |
| Proteasome | 24 | 0.596571 | 2.13289 | 0.000692 | 0.009204 |
| T cell receptor signaling pathway | 69 | -0.55981 | -1.65385 | 0.000728 | 0.009204 |
| Fc epsilon RI signaling pathway | 45 | -0.60146 | -1.69274 | 0.000758 | 0.009204 |
| Type II diabetes mellitus | 31 | -0.65234 | -1.74549 | 0.000902 | 0.009875 |
| Drug metabolism cytochrome p450 | 33 | 0.588501 | 2.305717 | 0.000929 | 0.009875 |
| Glutathione metabolism | 37 | 0.554533 | 2.231468 | 0.00105 | 0.009962 |
| B cell receptor signaling pathway | 57 | -0.57098 | -1.65511 | 0.001055 | 0.009962 |
| Cell adhesion molecules cams | 79 | -0.52597 | -1.57446 | 0.001337 | 0.011962 |
| Biosynthesis of unsaturated fatty acids | 13 | 0.68866 | 2.038448 | 0.001759 | 0.014952 |
| Terpenoid backbone biosynthesis | 11 | 0.722471 | 2.029395 | 0.001993 | 0.016132 |
| Porphyrin and chlorophyll metabolism | 18 | 0.583697 | 1.924994 | 0.002187 | 0.016165 |
| Steroid hormone biosynthesis | 18 | 0.585891 | 1.932228 | 0.002187 | 0.016165 |
| Metabolism of xenobiotics by cytochrome p450 | 31 | 0.484282 | 1.860679 | 0.002643 | 0.018722 |
| Dilated cardiomyopathy | 56 | -0.54624 | -1.58101 | 0.003379 | 0.022975 |
| Fc gamma R mediated phagocytosis | 63 | -0.52549 | -1.53905 | 0.004811 | 0.030028 |
| Nod-like receptor signaling pathway | 36 | -0.59225 | -1.61888 | 0.004882 | 0.030028 |
| Fatty acid metabolism | 30 | 0.470206 | 1.786837 | 0.005093 | 0.030028 |
| Allograft rejection | 10 | -0.77007 | -1.66359 | 0.005299 | 0.030028 |
| Pentose and glucuronate interconversions | 10 | 0.719714 | 1.950477 | 0.005299 | 0.030028 |
| Retinol metabolism | 24 | 0.509653 | 1.822135 | 0.005533 | 0.03034 |
| Protein export | 14 | 0.634547 | 1.931501 | 0.006413 | 0.03407 |
| Leukocyte transendothelial migration | 77 | -0.49977 | -1.49251 | 0.007007 | 0.034448 |
| JAK STAT signaling pathway | 71 | -0.50763 | -1.50512 | 0.007053 | 0.034448 |
| Ubiquitin mediated proteolysis | 80 | 0.315307 | 1.474865 | 0.007092 | 0.034448 |
| Steroid biosynthesis | 13 | 0.627846 | 1.858437 | 0.007916 | 0.037379 |
| Graft versus host disease | 11 | -0.73859 | -1.62677 | 0.008541 | 0.039244 |
| Valine leucine and isoleucine degradation | 35 | 0.410512 | 1.629779 | 0.009699 | 0.043392 |
| N glycan biosynthesis | 28 | 0.45657 | 1.712081 | 0.010228 | 0.044299 |
| Type I diabetes mellitus | 13 | -0.70736 | -1.61649 | 0.010611 | 0.044299 |
| Oxidative phosphorylation | 61 | 0.321093 | 1.426284 | 0.010684 | 0.044299 |
| Regulation of autophagy | 14 | 0.598489 | 1.821745 | 0.011452 | 0.046354 |

**Supplementary Table 10.** Gene Set Enrichment Analysis (GSEA) of KEGG pathway between MCD vs HGPP group (|NES|＞1 and *P* adjust＜0.05).

| KEGG pathway | Size | ES | NES | *P* value | *P* adjust |
| --- | --- | --- | --- | --- | --- |
| Cell cycle | 119 | 0.55761527 | 1.85392733 | 0.00011509 | 0.01073601 |
| Cell adhesion molecules cams | 93 | 0.5741544 | 1.85744329 | 0.00011929 | 0.01073601 |
| ErbB Signaling Pathway | 82 | 0.56303672 | 1.79073098 | 0.0002434 | 0.01460387 |
| Valine Leucine and Isoleucine Degradation | 41 | -0.652028 | -2.2028374 | 0.00039793 | 0.01482397 |
| Peroxisome | 75 | -0.5498875 | -2.1143717 | 0.00052438 | 0.01482397 |
| Leukocyte transendothelial migration | 101 | 0.519671 | 1.69414936 | 0.00058969 | 0.01482397 |
| Focal adhesion | 181 | 0.45448113 | 1.56635189 | 0.00065674 | 0.01482397 |
| Butanoate metabolism | 28 | -0.6431654 | -2.002387 | 0.00069711 | 0.01482397 |
| ECM receptor interaction | 75 | 0.55659252 | 1.74802186 | 0.0007412 | 0.01482397 |
| Drug metabolism other enzymes | 27 | -0.6472459 | -2.0011728 | 0.00102459 | 0.01788376 |
| Tryptophan metabolism | 34 | -0.5553653 | -1.8070458 | 0.0010929 | 0.01788376 |
| Lysine degradation | 43 | -0.5000976 | -1.7146351 | 0.0011976 | 0.01796407 |
| P53 signaling pathway | 59 | 0.57029244 | 1.73130644 | 0.00140593 | 0.01946673 |
| Cytokine receptor interaction | 172 | 0.44610869 | 1.53159776 | 0.00165107 | 0.02122808 |
| Steroid hormone biosynthesis | 24 | -0.6022841 | -1.7978182 | 0.0036888 | 0.04297721 |
| Glioma | 60 | 0.53922598 | 1.64063101 | 0.0038202 | 0.04297721 |

**Supplementary Table 11.** Gene Set Enrichment Analysis (GSEA) of KEGG pathway between MCS vs HGPP group (|NES|＞1 and *P* adjust＜0.05).


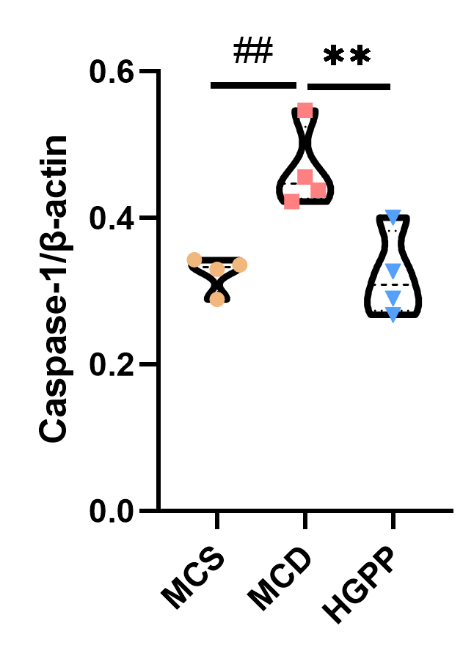
**
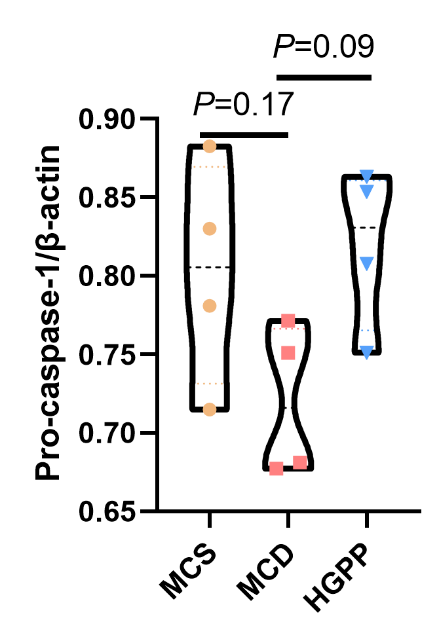
**

**Supplementary Figure 4.** The western blotting analysis of Caspase-1/β-actin and Pro-caspase-1/β-actin.
